# Spatial scale break in ecological strategies for host use by plant viruses

**DOI:** 10.3389/fmicb.2026.1797573

**Published:** 2026-05-07

**Authors:** Michael McLeish, Adrian Zamfir, Bisola Babalola, Miguel Ángel Mora, Aurora Fraile, Fernando García-Arenal

**Affiliations:** Centro de Biotecnología y Genómica de Plantas (CBGP UPM-CSIC/INIA), Universidad Politécnica de Madrid and E.T.S.I. Agronómica, Alimentaria y de Biosistemas, Campus de Montegancedo, UPM, Madrid, Spain

**Keywords:** coinfection, community, cooccurrence, disease risk, host competence, metaviromics, scaling domains, transmission

## Abstract

Processes that involve performance traits such as host range underlie pathogen diversity and infection risk. Host range performance (HRP) is expected to be distributed across spatially explicit conditions, but rarely considered in microbial ecology. Merging of spatially discrete processes in epidemiological model selection exacerbates error propagation. We combine high throughput sequencing and reverse transcriptase polymerase chain reaction approaches to test whether HRP of 18 plant virus species influences species diversity across spatial scales. The results indicated a scaling break in the effect of HRP between the individual host and community scales. Regression analyses showed weaker (i.e., random) associations between viruses in individual hosts than at broader spatial scales of cooccurrence among hosts. Contrasting variances in effect sizes between the spatial scales indicated HRP informs on disease risk at the plant community level, but is a poor predictor of infection in individual plants. The evidence of contrasting HRP between the domains of scale set in this study, suggests heterogeneity among multiple processes across scales drive virus species diversity. Epidemiological model selection should consider variation in species trait performance across scales.

## Introduction

Coexistence of pathogens is an outcome of trade-offs across hosts and the ability to spread among them (Tack et al., 2012; Elena and García-Arenal, 2023). The maintenance of pathogen populations is possible through multiple processes (e.g., selection, dispersal, stochasticity) that propagate at both local-population and larger spatial scales, and conditions for coexistence are therefore highly variable (Chesson, 1985; Thrall and Burdon, 1999; Ladau and Eloe-Fadrosh, 2019). However, hypotheses of scale-dependent processes that drive pathogen coexistence in natural communities remain undertested (Cohen et al., 2016; Kay et al., 2019; Kendig et al., 2024; Gilbert et al., 2025). For instance, pathogen cooccurrence can be in the same or different tissues of the hosts they infect (Seabloom et al., 2015), or among hosts of the communities they persist in (DiRenzo et al., 2018). Trait responses that underlie pathogen coexistence are expected to be distributed across widely dissimilar spatial scales (Adler et al., 2013). Host range is a performance trait (Poisot et al., 2011; DeLong et al., 2022) fundamental in the ability to spread and in the number of host resources that can be encountered and accessed (Thrall and Burdon, 1997; Fermin, 2018) and central to the epidemiology of diseases (Woolhouse et al., 2001; Lefeuvre et al., 2019). Host range performance (HRP) directly determines reproduction and survival, the two core components of fitness (DeLong et al., 2022). It is expected that host range links plant virus species performance with their cooccurrence under a number of spatially explicit conditions.

Scale dependencies have been recognised as a key component of ecology because they have an enormous effect on observations of pattern and process (Chase and Leibold, 2002). Changes in pattern due to non-linear interactions caused primarily by spatial heterogeneity, may indicate a scaling break and can be used to define domains of scale (Wu and Li, 2006). A domain of scale is defined as a portion of the scale spectrum within which process-pattern relationships are consistent regardless of scale (Wiens, 1989). A domain of scale will possess unique properties where patterns or processes are predicted to have a stronger effect size: i.e., statistics that measure the magnitude of effects (Nakagawa and Cuthill, 2007). Community ecology is a versatile approach in linking within-host processes with those at larger scales of the ecosystem (Johnson et al., 2015; Rynkiewicz et al., 2015; Lefeuvre et al., 2019; Stüwe et al., 2025).

**Figure 1:**
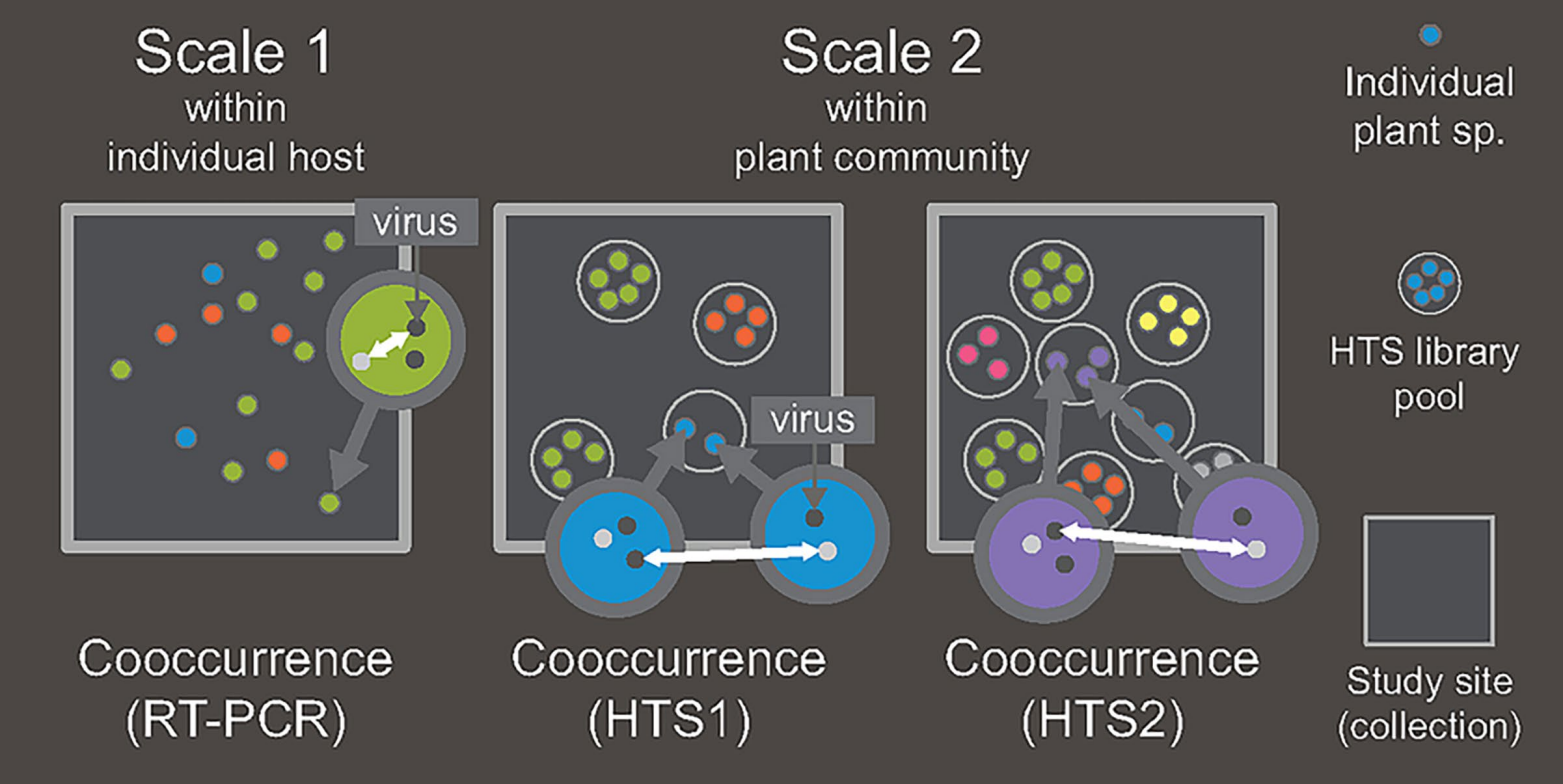
Study design showing the two scales at which cooccurrence was estimated. The scheme represents hypothetical study sites each for cooccurrence (double-headed white arrows). Scale 1 is where instances of cooccurrence within individual plants were detected with RT-PCR. Cooccurrence between viruses under Scale 2 (e.g., four HTS1 pools of samples of three plant species; i.e., green, red, blue) are between HTS libraries that comprise pools of individuals of a single species and collection sampled for HTS detection where the viruses were detected by RT-PCR. Dataset HTS2 includes additional libraries that were not subject to RT-PCR assays. Cooccurrences at Scale 2 are at a wider spatial scale than at Scale 1 because each pool consists of spatially discrete plant samples collected over the study site while at Scale 1 detections are from within an individual plant.

Here we elucidate on the spatial scale(s) at which observed host range (i.e., host range performance, HRP) influence generalist plant virus cooccurrence in natural communities. Patterns of plant virus diversity within and between plants, can be exploited to discriminate particular spatial scales at which processes act to determine infection distributions. We harness implicit spatial scale distinctions between coinfection within individual plants, and cooccurrence in plant communities of an ecosystem, to examine whether HRP acts across these scales of organisation (Figure 1). To do this we frame species interactions at two spatial scales and compare: (i) observations of cooccurrence based on high throughput sequencing (HTS) with; (ii) coinfection detected via reverse transcriptase polymerase chain reaction (RT-PCR). We then test whether HRP has an influence at either level of virus species organisation. Specifically, we test the null hypothesis that HRP operates on species cooccurrence, across domains of scale, both at the individual plant scale and the plant community scale.

## Materials and methods

### Study design and sample units

Plant collections were conducted between July 2015 and June 2017 in the Vega del Tajo-Tajuña region of the Tagus River Basin in the South-Central plateau of Spain. Briefly, collections were conducted at fixed distances of 1 m along random transits at each site regardless of the sample showing symptoms of infection or not, distance between plants from the same site ranged from 1 to 50 m (McLeish et al., 2024). Collections were conducted at multiple sites of each of four habitats under different degrees of disturbance: Oak forests (Oak) are native communities dominated by evergreen *Quercus* spp.; Wasteland includes undisturbed patchy areas of successional shrubland, with no use in the last ten years; Crop are habitats where annual monocultures share the space with wild plant species (weeds), and Edge are the narrow borders that separate crop fields, with plant communities that benefit from nutrient and water supplemented to adjacent crops. A total of 78 collections were done at four sites each of Oak, Wasteland and Edge and eleven sites of Crop, the higher number of sites representing the main crops of the area, maize (2 sites) and melon (4 sites) as summer crops, and barley (3 sites) and brassicas (2 sites) as winter crops. The extent of the study area is larger than 1,250 km^2^, and large enough to assume that migration in and out of the area not to affect community dynamics (Chesson, 2000). Rarefaction showed near asymptotic relationships between the number of samples and the expected number of species at each collection (McLeish et al., 2021). Total RNA extractions of individual plants from the same species and collection were pooled to create libraries for HTS reported in previous work (McLeish et al., 2022; McLeish et al., 2024). Local BLAST queries, version 2.2.29 (Camacho et al., 2009), of the HTS reads were conducted against a database of plant virus genomic references (https://www.ncbi.nlm.nih.gov/genomes/ accessed December 2018) to detect operational taxonomic units (OTUs), see supplementary material for details. The BLAST query matches were standardised across libraries to minimise both the frequency of false positives and the exclusion of true positive detections. Reads were retained when the query coverage was 100% and the alignment length was greater or equal to 125 nt. These two steps optimised sequence similarity between read and reference, while permitting mismatches expected from divergent genome sequences.

From a total of 235 OTUs detected with HTS, eighteen generalist plant viruses that occurred in at least three of the four habitats above (Table 1) were selected for this study where RT-PCR was conducted for each RNA extract of individual plant samples pooled to create the HTS libraries. The selection of the eighteen viruses was based on the distribution of their host ranges (Supplementary Figure 1), transmission mode, and general research interest (e.g., economic importance). There was a mean of 6 extracts (range from 1 to 48) per pool, whose abundance varied according to their relative abundance across the study sites.

**Table 1:** The eighteen viruses detected with the RT-PCR assay.

Genus	Family	NCBI title	Abbreviation	Genome
Polerovirus	Luteoviridae	Beet chlorosis virus	BChV	(+)ssRNA
Polerovirus	Luteoviridae	Beet mild yellowing virus	BMYV	(+)ssRNA
Luteovirus	Luteoviridae	Barley yellow dwarf virus	BYDV	(+)ssRNA
Polerovirus	Luteoviridae	Cucurbit aphid-borne yellows virus	CABYV	(+)ssRNA
Cucumovirus	Bromoviridae	Cucumber mosaic virus	CMV	(+)ssRNA
Tobamovirus	Virgaviridae	Pepper mild mottle virus	PMMoV	(+)ssRNA
Ilarvirus	Bromoviridae	Parietaria mottle virus	PMoV	(+)ssRNA
Potyvirus	Potyviridae	Plum pox virus	PPV	(+)ssRNA
Anulavirus	Bromoviridae	Pelargonium zonate spot virus	PZSV	(+)ssRNA
Sobemovirus	Solemoviridae	Rubus chlorotic mottle virus	RuCMV	(+)ssRNA
Cucumovirus	Bromoviridae	Tomato aspermy virus	TAV	(+)ssRNA
Carmovirus	Tombusviridae	Turnip crinkle virus	TCV	(+)ssRNA
Tobamovirus	Virgaviridae	Tobacco mild green mosaic virus	TMGMV	(+)ssRNA
Tobamovirus	Virgaviridae	Tobacco mosaic virus	TMV	(+)ssRNA
Potyvirus	Potyviridae	Turnip mosaic virus	TuMV	(+)ssRNA
Polerovirus	Luteoviridae	Turnip yellows virus	TuYV	(+)ssRNA
Potyvirus	Potyviridae	Watermelon mosaic virus	WMV	(+)ssRNA
Tobamovirus	Virgaviridae	Youcai mosaic virus	YoMV	(+)ssRNA

### Spatial scale analysis

We analyse cooccurrence at what we define as “Scale 1” and “Scale 2” to differentiate between ecological strategies of coexistence within a host plant and within a community, respectively. A general definition for coexistence is the ability of cooccurring species to persist (i.e., retain positive abundances) in a community across defined spatiotemporal scales (Clark et al., 2025). Previous work has shown that the plant virus species analysed in this study show patterns of habitat specificity among communities, where virus abundances and community maintenance is determined by habitat affiliation (Malpica et al., 2006; Valverde et al., 2020). For the purposes of this study we assume that plant virus distributions among host species are a function of two domains of scale either as coinfections (i.e., more than a single virus detected from an individual plant sample; no distinction with superinfection) within an individual host plant (i.e., Scale 1), or as infections across a host species of a plant community (i.e., Scale 2). Analyses were designed (Figure 1) to quantify: (i) frequencies of infection as determined by RT-PCR, detected from individual samples (e.g., Scale 1) that were pooled to make up each HTS library; or (ii) frequencies of infection across hosts detected from the same HTS pools above, between HTS libraries of the same species collected at the same study site and time (e.g., Scale 2).

The analysis implemented in the R package *cooccur* (Veech, 2013; Griffith et al., 2016), treats either the HTS sequencing library pool as “site” or the individual host plant sample as “site.” Frequencies of infection both within and between hosts were estimated from sample collections conducted at a given study site and time. Scale 1 comprised instances of infection within an individual plant that were detected with RT-PCR. The estimates of species infection among a host species at Scale 2 were based on detections between HTS libraries. We treated the HTS observations in two ways. First, we retained virus OTUs from the HTS libraries that were used in the RT-PCR assays (HTS1), and second, included additional host species of the same 18 viruses (HTS2) not represented in the RT-PCR assays (*n* = 310 libraries and *n* = 113 host spp.). The HTS2 were used to estimate HRP of each of the eighteen viruses. Cooccurrences at Scale 2 among spatially discrete plant samples collected over the study site are at a wider spatial scale than coinfections within an individual plant at Scale 1. Bartlett’s tests for homoscedasticity between the HTS and RT-PCR datasets were conducted to compare variance in effect sizes between scales. To visualise the connectivity and strength of effect sizes observed between the 18 viruses, unipartite networks, produced with the R package *igraph* (Csardi and Nepusz, 2006), and heatmaps were generated.

To account for unequal sample sizes between the HTS and RT-PCR observations, effect sizes estimated from the cooccurrence analyses were standardised (see below). Effect sizes are the differences between expected and observed frequency of cooccurrence between each virus pair. The values are standardised by dividing the difference by the number of sample units in each dataset. In standardised form, the values are bounded from −1 to 1. Significant positive values indicate greater than expected frequency of cooccurrence and significant negative values less than expected frequencies.

### Regression modelling

Host range describes the degree to which a parasite specialises on resources used to meet the requirements for reproduction and survival. Here, HRP is defined as the number of host species (*n* = 113 possible species) that a virus OTU was detected from. The influence of HRP on coexistence strategies was estimated at the two scale domains (Figure 1) based on either the RT-PCR or HTS observations from 21 sites across the extent of the study (Supplementary Figure 2).

Linear regression models were designed to examine the effect of independent variables, host range mean and host range difference, on the response as standardised effect sizes of cooccurrences. As effect sizes are based on the relationship between pairs of taxa, the two host range indices were created for the regression modelling to reflect the pair-wise cooccurrence associations. The mean host range (the host range mean of virus pair *a* and *b*) provides an estimate of the host range breadth of both viruses of a pair, while the difference (the absolute difference value between the host range of virus pair *a* and *b*) represents a contrast in breadth between virus pairs. We assume that both indices represent components HRP that are relative to each virus pair, the former representing relative performance similarity and the latter, relative performance differences. The relationship between the dependent variable and the independent variable was examined with a regression coefficient test, implemented with heteroscedasticity-consistent estimation of the covariance matrix, conducted with the R package *sandwich*.

## Results

### RT-PCR detections

The Complete HTS data resulted in *n =* 235 OTUs detected from 310 libraries (235 × 310 matrix) of 113 host species. The eighteen viruses detected with RT-PCR are shown embedded in a unipartite network of interactions (Supplementary Figure 3) with another 217 OTUs detected from sites across the extent of the study. The HTS libraries in which the eighteen viruses were detected by RT-PCR produced a subset of samples from 36 collections of 21 study sites. From the 2,017 RT-PCRs that were conducted with individual plant samples, 717 complete records were recovered for all eighteen viruses. We estimated effect sizes from the HTS1 dataset that comprised 118 libraries that were used in the RT-PCR assays, representing 60 host species, and also with additional libraries of host species of the same 18 viruses, not represented in the RT-PCR assays (HTS2) that comprised 310 libraries of 113 host species of the 18 focal virus OTUs. Three viruses in particular had lower frequencies of links with other viruses under coinfection at Scale 1 as estimated from the RT-PCR assays, compared to infection among hosts at Scale 2 (Supplementary Table 1). Plum pox virus (PPV), turnip crinkle virus (TCV), and barley yellow dwarf virus (BYDV) had relatively low node degrees (*k*) at Scale 1, which indicated distinct ecological strategies for resource use resulting in cooccurrence with other virus species in a community being more likely than in an individual plant.

### Spatial scale analysis

The RT-PCR dataset produced 717 individual plant samples (60 host species) that were infected by one or more of the eighteen focal viruses. The matrix used for the cooccurrence analysis at Scale 1 (within a host) had the dimensions 18 × 717. Of the possible 153 virus-virus pairs, 116 (75.82%) were detected as 1,385 coinfections (Supplementary Table 2; Supplementary Figure 4). The HTS datasets used to analyse cooccurrence of the 18 OTUs at Scale 2 among hosts resulted in: (i) an 18 × 118 matrix of 118 libraries that comprised 60 host species with 2,257 cooccurrences (HTS1); and (ii) an 18 × 310 matrix of 310 libraries that represented 113 host species with 3,458 cooccurrences (HTS2). Of the 18 OTUs detected with HTS1, there were 139 virus-virus pair types (90.85% of possible pairs) (Supplementary Table 3; Supplementary Figure 4), and from the HTS2 dataset, there were 146 virus-virus pair types (95.42% of possible pairs) (Supplementary Table 4; Supplementary Figure 4). Note that cooccurring pairs of viruses represent non-random associations in a probabilistic test, with no assumption of mechanisms behind these cooccurrences.

**Figure 2:**
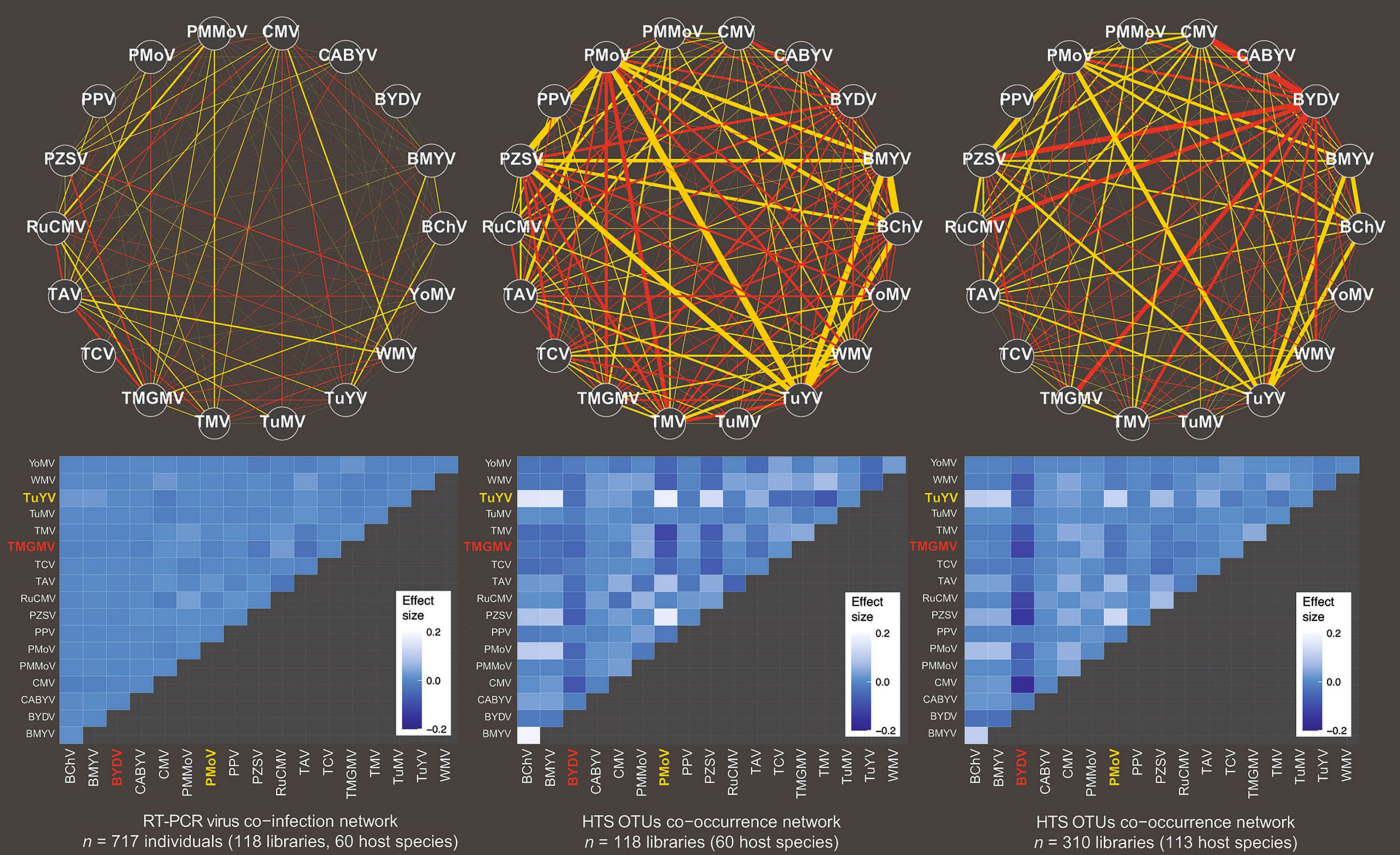
Unipartite networks and heatmaps of the eighteen viruses (left panel), or OTU equivalents (central and right panels), and effect sizes of cooccurrence frequencies. Networks and heatmaps are for analyses at scale 1 (left), and at scale 2 HTS1 (centre) or HTS2 (right). Weights of network links (negative in red, positive in green) are proportional to the effect size, and the size of the nodes are proportional to the number of nodes they are connected to. Some network interactions highlighted on heatmap in green and red text.

**Figure 3:**
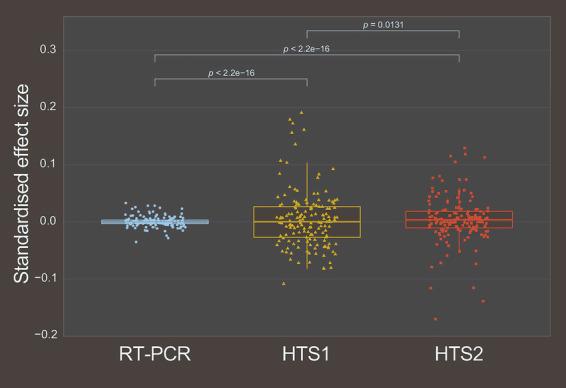
The relationships between the standardised effect sizes of the RT-PCR, HTS1, and HTS2 datasets. Significance of Bartlett’s test of homogeneity of variances shown with hairs at the top of the plot.

Standardised effect sizes (i.e., the magnitude of the effect of cooccurrence frequency) were calculated for all three datasets above (Supplementary Table 5). Unipartite networks and heatmaps were generated in each case to visualise the differences in connectivity and effect sizes of the cooccurrence frequencies (Figure 2). The heatmaps of effect sizes indicated differences in cooccurrence patterns between the datasets. Effect sizes were in general smaller with less variance at Scale 1 within hosts compared to both HTS1 and HTS2 cooccurrences among hosts at Scale 2. Of the latter two datasets, HTS1 had more extreme positive effect sizes and less extreme negative effect sizes than the HTS2 cooccurrences (Figure 3). Bartlett’s tests indicated significant variances among all three, but relatively similar between the HTS datasets. The distinct patterns among the variance distributions of effect size were consistent with the datasets being related to a particular domain of scale. Variation was greatest between the cooccurrences at Scale 2 compared to Scale 1 (Figure 1), which indicated weaker (random) associations between viruses under coinfection than at broader spatial scales among host plants of a community. In other words, some virus cooccurrences are better predicted in a given community, but not in a given individual plant in that community. The variation in effect sizes of the HTS2 dataset with the addition of host species/libraries, indicated a positive relationship with negative effect size values indicative of less than expected frequency of cooccurrence. In all, cooccurrence at Scale 2 was more likely a function of the virus pair than under coinfection at Scale 1. The greatest disturbances to virus-pair ranks of effect size occurred between the RT-PCR and either of the HTS estimates, which indicated a break to coexistence patterns between the two scales of organisation (Supplementary Figures 5, 6).

Cooccurrence analyses also showed a number of significant probabilities (α = 0.05) where two viruses coinfected (RT-PCR) or cooccurred (HTS1 & HTS2) more or less than expected by chance (Supplementary Table 6). Overall, the results show fewer instances (21%) of significant (either more or less than expected) coexistence at both Scales 1 and 2. We refer to significant interactions that are indicative of either ecological limitations on cooccurrence, or ecological factors that promote cooccurrence as a “coexistence strategy” (Supplementary Table 7). Of the 153 possible virus-virus pair combinations, *n* = 47 produced no significant across-scale or scale-specific strategy for coexistence (i.e., no significant cooccurrence). There were *n* = 8 instances of contrasting strategies across-scales; i.e., significantly positive or negative at one scale, with the opposite sign pattern at the other scale. The remaining *n* = 98 virus pairs showed uniform strategies either specific to a scale (*n* = 66) or across scales (*n* = 32). In agreement with the weak effect sizes with hosts, there were *n* = 5 significant positive (and 10 significant negative) virus pair associations at Scale 1, compared to 22 positive and 29 negative associations at Scale 2. With care taken not to overinterpret the prevalence of each of these categories, the results show distinct coexistence strategies in respect to the spatial scales.

As Scale 2 restricted the breadth of resource available to each virus pair to a single host species, we were interested in assessing effect sizes when the number of host species was increased to greater than one. We aggregated the interaction matrix by collection to assess cooccurrence with the addition of host species available to each possible virus pair. In effect, this increased the possibility of coexistence among different host species (i.e., niche/resource availability), but does not necessarily translate to a change in spatial scale among infections. With additional hosts, the effect sizes among virus pairs increased (Supplementary Figures 7, 8), where larger host ranges predicted stronger effect sizes in coexistence among virus pairs.

### Regression modelling

We used linear regression to test the relationship between HRP and effect sizes of the RT-PCR, HTS1, and HTS2 datasets (Table 2). Host range mean and difference had no significant effect on effect sizes of cooccurrence at Scale 1, but had varying significant effects at Scale 2. Host range difference had a significant (*β* = − 0.001, *p* = 0.0415) influence on effect sizes of the HTS1 cooccurrence dataset, while both host range mean (*β* = 0.001, *p* < 0.0001) and difference (*β* = − 0.001, *p* = 0.005) were significant predictors of effect size with the HTS2 data. Dissimilarity in HRP between virus pairs was a significant predictor under cooccurrence, while similarities in HRP were significant only when we included the complete set of 113 host species from which the OTUs were detected. The HTS2 data produced the only overall significant (Table 3) model (adj. *R*^2^ = 0.113, *F*_(2, 150)_ = 10.67, *p* < 0.0001). Together, the regression models indicate that differences in HRP was a predictor of cooccurrence at Scale 2. Similarities in host range was a predictor only at Scale 2 in the local host species community of a given study site. The negligible influence of the host range predictors on coinfections indicated a contrasting pattern of cooccurrence between Scale 1 and 2.

**Table 2:** Regression model tests of coefficients for the effects of host range mean and difference on standardised effect sizes of cooccurrence.

	Coefficients	Estimate	SE	*t*	*p*-value
RT-PCR (Scale 1)	Intercept	5.1E−04	2.1E−03	2.4E−01	0.8095
	H.R. mean	5.3E−05	6.7E−05	7.8E−01	0.4341
	H.R. difference	−8.7E−05	4.4E−05	−2.0E+00	0.0511
HTS1 (Scale 2)	Intercept	−0.002	0.009	−0.207	0.8364
	H.R. mean	0.000	0.000	1.771	0.0786
	H.R. difference	−0.001	0.000	−2.056	**0.0415**
HTS2 (Scale 2)	Intercept	−0.013	0.008	−1.627	0.1059
	H.R. mean	0.001	0.000	4.031	**8.8E−05**
	H.R. difference	−0.001	0.000	−2.874	**0.0046**

The regressions were conducted with the RT-PCR cooccurrence frequencies at Scale 1 and the HTS1 and HTS2 cooccurrence frequencies at Scale 2. Significant *p*-values shown in bold text.

**Table 3:** Linear regression model *F*-statistics for the overall relationship between the effect size responses and independent variables for host range mean and difference.

	Adj. *R*^2^	SE	*F*-statistic	DF	*p*-value
RT-PCR	0.011	0.009	1.837	150	0.1629
HTS1	0.020	0.050	2.553	150	0.0813
HTS2	0.113	0.039	10.670	150	**4.6E−05**

Significant model shown in bold.

## Discussion

Spatial scales of ecological processes are important because they shape species trait variation and coexistence (Wiens, 1989; Kneitel and Chase, 2004; Laine et al., 2011; Thrall et al., 2016; McLeish et al., 2017; Biswas et al., 2024). Identifying the scales that capture variation of species performance traits is a necessary step in minimising error propagation across scales and in epidemiological model selection (Cunniffe et al., 2015; Jeger et al., 2018; Newman et al., 2019), and towards the eventual goal of forecasting disease risk (Tack et al., 2014; McLeish et al., 2020). The spatial distribution of infection at ecosystem scales, has been shown to be strongly structured by coinfection, a space where pathogens coexist at small spatial scales. Coinfection of a host by multiple viruses is common (Tollenaere et al., 2016; Díaz-Muñoz, 2017; Norberg et al., 2023) and has consequences for their survival, transmission, and evolution (Elena et al., 2014a,b). For example, within-host accumulation of pathogens has positive and negative influences on transmission in coinfected hosts and is hypothesised to affect host range evolution (Seabloom et al., 2009; Antonovics et al., 2017). At larger regional scales among plant communities, greater than expected frequent encounters among specific subsets of viruses and hosts were a product of coinfection, which suggested HRP was a function of processes that transition across scales (McLeish M. et al., 2019). In short, pathogen multiplication and transmission traits are affected by spatial structuring among communities (Messinger and Ostling, 2009), which highlights the necessity in determining the spatial scales that structure viral resource exploitation strategies (Thrall and Burdon, 1997; Berngruber et al., 2015).

Spatial scale thresholds for the influence of plant virus host range on infection distributions and coexistence in natural systems is largely unknown. Importantly, these thresholds are not known for other pathogen groups (Seabloom et al., 2015; Sieben et al., 2022). Here we assume that virus species coexistence is organised across two domains of scale represented by cooccurrences either as coinfections within individual hosts or across hosts in plant communities (or Scales 1 and 2, respectively, Figure 1). We test the null hypothesis that host range affects species cooccurrence across these scales of spatial organisation. When significant instances of all 153 virus-pair combinations (Supplementary Table 6) were collapsed into categories (Supplementary Table 7), or “ecological strategies”, most pairs exhibited relationships defined by a scaling break between Scales 1 and 2. Changes to the rank of effect size between particular virus pairs (Supplementary Figure 5), the correlations among effects sizes observed at each Scale (Supplementary Figure 6), and in the proportion of ecological strategies (Supplementary Table 7) specific to a scale (*n* = 66) and across scales (*n* = 32), were all indicative of a break in spatial domains of species cooccurrence. There were also 8 additional instances of contrasting strategies across scales where a virus pair was observed at a frequency greater than expected by chance at one spatial scale, and less than in the other. Host range dissimilarities between virus pairs were a stronger predictor of effect sizes at Scale 2, with host range similarities between virus pairs significant only when additional host species were included (HTS2) in the analysis (Table 2). The findings support an effect of a scaling break between Scale 1 and 2, and by extension, a threshold for a break in processes, including HRP, which drive species cooccurrence associated with each scale.

The variation in effect sizes and significantly greater or less than expected frequencies of cooccurrence of particular virus-pair combinations, coincided with the change in spatial scale. Although the domains of scale used in the analyses are intuitive, if not obvious, the evidence for the limitations of HRP on infection distributions is a fruitful gain. It can be hypothesised that breaks in the patterns between the scales demonstrated here, also coincide with, or approximate, other factors relevant to particular spatial scales in virus species ecological strategies (Supplementary Table 7). Distinctions associated with each domain of scale shown here, can be framed by transmission among hosts and the spatial distribution of host susceptibility to infection, and during infection by host defence mechanisms and factors in the competence to transmit.

Coexistence of viruses is known to be strongly influenced by plant community composition (Bernardo et al., 2018), the distribution of plant traits (Laine et al., 2011), and interactions with vectors (Blanc et al., 2014) and virus interactions during infection (Elena et al., 2014a; Hily et al., 2016). At the plant community level of organisation, the ecology of the eighteen viruses is known to be a consequence of habitat specificity (Valverde et al., 2020) and host selectivity (Malpica et al., 2006), which each encompass numerous processes. In many instances the movement ecology of viruses is dependent on insect vectors, or on one of several other modes of transmission via contact and seed (Mauck et al., 2012; Roossinck, 2013; Hamelin et al., 2016), and ecological compatibilities that depend on plant community composition (Peláez et al., 2020). For instance, viruses will tend to infect host species that occur in multiple plant communities, but display stronger affiliations with a host in a given habitat due to the plant community composition (Bernardo et al., 2018; Susi and Laine, 2021; Norberg et al., 2023; McLeish et al., 2024). Therefore, a suite of possible traits involved with host resource use by viruses, which respond to the biotic and abiotic environment, partly determine HRP at a landscape scale. The diversity of mechanisms that potentially shape host range performance (as opposed to host range evolution), the degree of stochasticity in virus-vector and virus-host interactions, predicts plastic, or multiple fitness components, would be advantageous (DeLong et al., 2022) for viruses to function in variable ecological circumstances, sufficient to allow population maintenance. For instance, the availability of multiple hosts and/or multiple vectors to viruses provide redundancies in transmission mechanisms (Power and Flecker, 2003). Trade-offs among performance traits (Tilman, 2000), habitat characteristics (Kraft et al., 2015), and neutral processes (Hubbell, 2005) are invoked to explain species distributions in general. Corresponding mechanisms have been acknowledged for pathogens of plants (Syller, 2012; Seabloom et al., 2013; Elena et al., 2014b; Norberg et al., 2023), and appreciated as crucial to disease ecology (Fussmann et al., 2007; Roossinck, 2013; Kay et al., 2019; Kendig et al., 2024). Multiple processes and mechanisms are required to explain the distribution of a virus at landscape levels of organisation. The limitations of virus HRP on cooccurrence below the plant community level, requires consideration of virus fitness components necessary for functioning at scales within hosts during infection.

Infection is a consequence of genetic mechanisms and fitness trade-offs involving both virus (Malpica et al., 2006; Syller, 2012) and host (Woolhouse et al., 2002; García-Arenal and Fraile, 2013) genotypes. Processes of infection involve facilitative and antagonistic virus-virus interactions (Syller and Grupa, 2016), mutation (Hillung et al., 2014), and recombination of genetic material (Lefeuvre and Moriones, 2015). Host competence (Cronin et al., 2010), sometimes referred to as host infectivity (i.e., the ability of an infected individual to transmit) (Tsairidou et al., 2019), is a key trait that has consequences for transmission due to the diversity of host phenotypes and symbiotic interactions potentially available within hosts (Tollenaere et al., 2016). For example, accumulation and transmission efficiency of tomato chlorosis virus (ToCV) and tomato infectious chlorosis virus (TICV) that coinfected *Physalis wrightii* and *Nicotiana benthamiana* hosts, resulted in changes in virus-specific titre that were host dependent (Wintermantel et al., 2008). Mixed patterns in plant-virus infectivity matrices shown by others (Moury et al., 2017; Valverde et al., 2020) might be explained by variation in host plant specialisation (McLeish et al., 2022), variation in infectivity and resistance phenotypes (Tack et al., 2012), or a degree of merging of spatially discrete processes (i.e., variables) among scaling domains that structure these interactions.

Scaling domains do not have to be structured among processes that are completely exclusive of one another, and some processes may broach the scaling break between other processes (Wiens, 1989). For instance, the analysis of *Linum marginale* infected by *Melampsora lini*, and *Plantago lanceolata* by *Podosphaera plantaginis* showed variation in resistance that declined towards larger spatial scales from individual hosts to among populations (Laine et al., 2011). This suggested that although disease resistance phenotypes were hierarchically structured across spatial scales, unexplained contributions of adaptive and non-adaptive (e.g., random) processes did not necessarily coincide with resistance structuring. Another example is when infection alters the perception of vectors towards host phenotypes at spatial scales wider than the individual. For instance, volatiles emitted from *Cucurbita pepo* plants infected by cucumber mosaic virus (CMV) were up-regulated and increased their apparency to aphid vectors relative to uninfected plants (Mauck et al., 2014). The mutations that promote mutualistic or antagonistic interactions within plants (Bedhomme et al., 2012; Elena et al., 2014a; Hily et al., 2016) are meaningful at the scale of the host individual, but have follow-in effects for transmission efficiencies (Wintermantel et al., 2008). Infection of hosts is a result of heterogeneous as well as multiscale transmission processes (VanderWaal and Ezenwa, 2016). Infection distributions and HRP are therefore emergent properties (Newman et al., 2019) of lower scale processes and not apparent from within-host patterns, which are instead observed at the among-host scale of organisation (McLeish M. et al., 2019). The mechanisms or processes that emerge as HRP are expected to be structured by multiple domains of scale. Several processes may overlap the scaling breaks that are distinguished by effect size thresholds of other processes. The structuring of domains of scales are also expected to be dependent on characteristics of the pathosystem (Elena, 2017) they are observed in.

We have shown that generalist plant viruses exhibit a number of ecological strategies that are organised in respect to scaling domains. HRP in natural environments was a better predictor of virus cooccurrence at spatial scales larger than the individual plant level. HRP is expected to be linked to processes of transmission where variation in encounters with host phenotypes, and in many cases with vectors, and stochastic factors that together contribute to virus species coexistence at the community and individual levels. The processes expected to contribute to plant virus coexistence involve selection via species interactions and environmental filters (e.g., niche differentiation), neutral stochastic colonisation and extinction events (Seabloom et al., 2013), and dispersal limitation (Johnson et al., 2015). However, heterogeneity in these processes of species coexistence are rarely considered in microbial ecology (Ladau and Eloe-Fadrosh, 2019) and disease ecology (Kendig et al., 2024), and remain a central consideration in ecology in general (Estes et al., 2018). Epidemiological processes that organise pathogen incidence and risk are subject to scale dependencies that have been acknowledged for some time (Ostfeld et al., 2005; Laine et al., 2011; Thrall et al., 2016; McLeish M. J. et al., 2019). The present findings suggest that plant virus HRP informs on disease risk at a scale of organisation compatible with the plant community. By extension, prediction of infection based on HRP at the scale of individual plants, is expected to be unreliable for generalist pathogens. Furthermore, the evidence suggests that HRP of generalist pathogens is unlikely to be a strong predictor of disease risk within a community at the host species level of organisation, when alternative hosts are available. The present study has been done in a limited geographical area, and more studies in other regions and involving other viruses will be needed to assess the generality of the findings. Although caution is required not to overinterpret the function of the hypothesised ecological strategies presented here, the findings provide a framework for categorising strategies and thresholding ecological processes in respect to spatial dependencies. Infection distributions observed at wide scales of organisation are emergent properties of finer scale processes where these patterns are not apparent. Future work on modelling virus distributions needs to consider the relationships between effect size of key processes and the scales at which they are most meaningful.

## Data Availability

All datasets presented in this study can be found in the article/Supplementary material.
