# Supplementary material for: Spatial scale break in ecological strategies for host use by plant viruses
Source: Front Microbiol. 2026 May 7;17:1797573. doi: 10.3389/fmicb.2026.1797573 (PMC13190531; doi:10.3389/fmicb.2026.1797573)
Supplement: Supplementary file 1 [file Supplementary_file_1.DOCX]

Supplementary Material

1. Supplementary methods

Total RNA extracted from individuals of the same plant species and collection were pooled to obtain a single HTS library. Both read orientations of each pool were run in the same sequencing lane, and each pool run in a separate lane. All libraries included a step for rRNA depletion using the RiboZero kit. Library preparation and high throughput sequencing with Illumina HiSeq platforms were as in (McLeish *et al.* 2022). Detection of operational taxonomic units (OTUs) of viruses was as in (McLeish *et al.* 2024). Local BLAST (i.e., the *blastn* function) queries were conducted with BLAST+ version 2.2.29 (Camacho et al. 2009) to detect virus OTUs from the high throughput sequence libraries. E-values are known to over- and under-estimate the significance of alignment especially in light of sequence database complexity. Rather than setting such a threshold, we implemented an *in silico* selection procedure that did not rely on the number of reads detected to verify the credibility of OTUs. The queries were conducted against plant virus genomic references of positive- and negative-sense single-stranded RNA (ssRNA), double-stranded RNA (dsRNA), and double- and single-stranded DNA (dsDNA and ssDNA) available from NCBI Viral Genome Browser (https://www.ncbi.nlm.nih.gov/genomes/ accessed December 2018). To decrease the frequency of false positive detections and minimize the exclusion of true positive virus OTUs that may result from database complexity that results from large intraspecific sequence divergence with a reference, and to standardise the BLAST query matches across libraries, reads were retained (i.e., OTUs detected) through the following pipeline only if: (1) the query coverage was 100 per cent; and (2) the alignment length was greater or equal to 125 nt.

# 2 Supplementary Figures and Tables

**Table 1.** Network node degree (*k*) for all virus-virus interactions under the three datasets. Node degree indicated the number of other virus species or OTUs each one cooccurred with at Scale 1 (RT-PCR) or Scale 2 (HTS1 & HTS2).

| **Virus/OTU** | **RT-PCR *k*** | **HTS1 *k*** | **HTS2 *k*** | **Mean** | **SD** |
| --- | --- | --- | --- | --- | --- |
| BChV | 13 | 15 | 15 | 14.33 | 1.15 |
| BMYV | 14 | 17 | 17 | 16.00 | 1.73 |
| BYDV | 7 | 10 | 16 | 11.00 | 4.58 |
| CABYV | 13 | 16 | 16 | 15.00 | 1.73 |
| CMV | 17 | 17 | 17 | 17.00 | 0.00 |
| PMMoV | 16 | 16 | 17 | 16.33 | 0.58 |
| PMoV | 11 | 17 | 17 | 15.00 | 3.46 |
| PPV | 7 | 10 | 11 | 9.33 | 2.08 |
| PZSV | 15 | 17 | 17 | 16.33 | 1.15 |
| RuCMV | 15 | 16 | 17 | 16.00 | 1.00 |
| TAV | 15 | 16 | 16 | 15.67 | 0.58 |
| TCV | 6 | 14 | 16 | 12.00 | 5.29 |
| TMGMV | 16 | 17 | 17 | 16.67 | 0.58 |
| TMV | 12 | 16 | 17 | 15.00 | 2.65 |
| TuMV | 15 | 16 | 16 | 15.67 | 0.58 |
| TuYV | 13 | 17 | 17 | 15.67 | 2.31 |
| WMV | 14 | 16 | 17 | 15.67 | 1.53 |
| YoMV | 13 | 15 | 16 | 14.67 | 1.53 |
|  |  |  |  |  |  |

**Table 2.** Observed number of cooccurrences from the RT-PCR dataset.

| RT-PCR | **BMYV** | **BYDV** | **CABYV** | **CMV** | **PMMoV** | **PMoV** | **PPV** | **PZSV** | **RuCMV** | **TAV** | **TCV** | **TMGMV** | **TMV** | **TuMV** | **TuYV** | **WMV** | **YoMV** |
| --- | --- | --- | --- | --- | --- | --- | --- | --- | --- | --- | --- | --- | --- | --- | --- | --- | --- |
| **BChV** | 10 | 2 | 3 | 2 | 3 | 1 | 0 | 4 | 4 | 6 | 0 | 5 | 0 | 4 | 18 | 4 | 0 |
| **BMYV** |  | 1 | 5 | 2 | 4 | 2 | 0 | 3 | 6 | 9 | 0 | 3 | 0 | 1 | 19 | 4 | 1 |
| **BYDV** | - |  | 0 | 1 | 0 | 0 | 0 | 0 | 0 | 1 | 0 | 1 | 0 | 1 | 3 | 0 | 0 |
| **CABYV** | - | - |  | 8 | 3 | 1 | 0 | 10 | 9 | 5 | 0 | 1 | 0 | 1 | 2 | 5 | 3 |
| **CMV** | - | - | - |  | 29 | 2 | 7 | 47 | 43 | 66 | 8 | 98 | 35 | 11 | 4 | 49 | 30 |
| **PMMoV** | - | - | - | - |  | 3 | 7 | 17 | 38 | 8 | 1 | 33 | 24 | 4 | 5 | 12 | 4 |
| **PMoV** | - | - | - | - | - |  | 0 | 8 | 0 | 17 | 0 | 0 | 4 | 1 | 2 | 2 | 0 |
| **PPV** | - | - | - | - | - | - |  | 6 | 15 | 0 | 0 | 9 | 9 | 0 | 0 | 0 | 1 |
| **PZSV** | - | - | - | - | - | - | - |  | 26 | 30 | 0 | 20 | 12 | 4 | 8 | 17 | 1 |
| **RuCMV** | - | - | - | - | - | - | - | - |  | 11 | 2 | 71 | 29 | 13 | 5 | 11 | 12 |
| **TAV** | - | - | - | - | - | - | - | - | - |  | 0 | 27 | 5 | 21 | 17 | 38 | 9 |
| **TCV** | - | - | - | - | - | - | - | - | - | - |  | 4 | 1 | 0 | 0 | 0 | 2 |
| **TMGMV** | - | - | - | - | - | - | - | - | - | - | - |  | 35 | 14 | 6 | 22 | 40 |
| **TMV** | - | - | - | - | - | - | - | - | - | - | - | - |  | 6 | 0 | 1 | 4 |
| **TuMV** | - | - | - | - | - | - | - | - | - | - | - | - | - |  | 3 | 5 | 2 |
| **TuYV** | - | - | - | - | - | - | - | - | - | - | - | - | - | - |  | 5 | 0 |
| **WMV** | - | - | - | - | - | - | - | - | - | - | - | - | - | - | - |  | 6 |
|  |  |  |  |  |  |  |  |  |  |  |  |  |  |  |  |  |  |

**Table 3.** Observed number of cooccurrences from the HTS1 dataset.

| HTS1 | **BMYV** | **BYDV** | **CABYV** | **CMV** | **PMMoV** | **PMoV** | **PPV** | **PZSV** | **RuCMV** | **TAV** | **TCV** | **TMGMV** | **TMV** | **TuMV** | **TuYV** | **WMV** | **YoMV** |
| --- | --- | --- | --- | --- | --- | --- | --- | --- | --- | --- | --- | --- | --- | --- | --- | --- | --- |
| **BChV** | 35 | 4 | 8 | 32 | 10 | 32 | 0 | 35 | 18 | 18 | 0 | 17 | 7 | 6 | 36 | 9 | 2 |
| **BMYV** |  | 9 | 8 | 36 | 11 | 34 | 1 | 38 | 19 | 20 | 1 | 18 | 8 | 6 | 38 | 10 | 2 |
| **BYDV** | - |  | 2 | 13 | 0 | 12 | 0 | 12 | 0 | 10 | 0 | 6 | 0 | 2 | 11 | 0 | 0 |
| **CABYV** | - | - |  | 9 | 5 | 8 | 0 | 9 | 9 | 6 | 2 | 7 | 4 | 3 | 9 | 7 | 2 |
| **CMV** | - | - | - |  | 31 | 55 | 7 | 70 | 40 | 47 | 15 | 53 | 34 | 13 | 50 | 37 | 12 |
| **PMMoV** | - | - | - | - |  | 15 | 6 | 23 | 22 | 10 | 6 | 24 | 16 | 3 | 15 | 15 | 5 |
| **PMoV** | - | - | - | - | - |  | 1 | 63 | 28 | 36 | 3 | 25 | 8 | 8 | 51 | 13 | 3 |
| **PPV** | - | - | - | - | - | - |  | 5 | 6 | 0 | 0 | 6 | 5 | 0 | 1 | 2 | 0 |
| **PZSV** | - | - | - | - | - | - | - |  | 39 | 39 | 3 | 38 | 18 | 10 | 54 | 21 | 6 |
| **RuCMV** | - | - | - | - | - | - | - | - |  | 15 | 7 | 30 | 21 | 4 | 24 | 22 | 8 |
| **TAV** | - | - | - | - | - | - | - | - | - |  | 7 | 23 | 14 | 7 | 30 | 20 | 2 |
| **TCV** | - | - | - | - | - | - | - | - | - | - |  | 11 | 10 | 5 | 3 | 12 | 7 |
| **TMGMV** | - | - | - | - | - | - | - | - | - | - | - |  | 27 | 7 | 26 | 26 | 9 |
| **TMV** | - | - | - | - | - | - | - | - | - | - | - | - |  | 6 | 9 | 22 | 9 |
| **TuMV** | - | - | - | - | - | - | - | - | - | - | - | - | - |  | 8 | 7 | 3 |
| **TuYV** | - | - | - | - | - | - | - | - | - | - | - | - | - | - |  | 12 | 3 |
| **WMV** | - | - | - | - | - | - | - | - | - | - | - | - | - | - | - |  | 9 |
|  |  |  |  |  |  |  |  |  |  |  |  |  |  |  |  |  |  |

**Table 4.** Observed number of cooccurrences from the HTS2 dataset.

| HTS2 | **BMYV** | **BYDV** | **CABYV** | **CMV** | **PMMoV** | **PMoV** | **PPV** | **PZSV** | **RuCMV** | **TAV** | **TCV** | **TMGMV** | **TMV** | **TuMV** | **TuYV** | **WMV** | **YoMV** |
| --- | --- | --- | --- | --- | --- | --- | --- | --- | --- | --- | --- | --- | --- | --- | --- | --- | --- |
| **BChV** | 43 | 12 | 9 | 37 | 12 | 37 | 0 | 41 | 21 | 20 | 0 | 22 | 10 | 6 | 44 | 9 | 2 |
| **BMYV** |  | 20 | 9 | 41 | 12 | 39 | 1 | 44 | 23 | 21 | 1 | 21 | 11 | 7 | 48 | 10 | 2 |
| **BYDV** | - |  | 17 | 37 | 13 | 25 | 0 | 35 | 49 | 16 | 3 | 31 | 23 | 2 | 29 | 9 | 2 |
| **CABYV** | - | - |  | 12 | 6 | 10 | 0 | 12 | 16 | 6 | 2 | 8 | 8 | 3 | 11 | 10 | 2 |
| **CMV** | - | - | - |  | 52 | 74 | 9 | 124 | 93 | 57 | 30 | 93 | 76 | 20 | 58 | 68 | 19 |
| **PMMoV** | - | - | - | - |  | 18 | 8 | 36 | 39 | 11 | 9 | 42 | 30 | 5 | 17 | 21 | 7 |
| **PMoV** | - | - | - | - | - |  | 1 | 85 | 44 | 39 | 3 | 29 | 17 | 8 | 57 | 18 | 3 |
| **PPV** | - | - | - | - | - | - |  | 8 | 7 | 0 | 1 | 10 | 7 | 0 | 1 | 3 | 0 |
| **PZSV** | - | - | - | - | - | - | - |  | 96 | 46 | 5 | 68 | 42 | 13 | 62 | 36 | 12 |
| **RuCMV** | - | - | - | - | - | - | - | - |  | 22 | 13 | 55 | 42 | 7 | 32 | 38 | 12 |
| **TAV** | - | - | - | - | - | - | - | - | - |  | 9 | 26 | 17 | 7 | 31 | 24 | 3 |
| **TCV** | - | - | - | - | - | - | - | - | - | - |  | 20 | 15 | 6 | 3 | 16 | 10 |
| **TMGMV** | - | - | - | - | - | - | - | - | - | - | - |  | 58 | 10 | 35 | 38 | 16 |
| **TMV** | - | - | - | - | - | - | - | - | - | - | - | - |  | 10 | 15 | 38 | 12 |
| **TuMV** | - | - | - | - | - | - | - | - | - | - | - | - | - |  | 9 | 11 | 3 |
| **TuYV** | - | - | - | - | - | - | - | - | - | - | - | - | - | - |  | 14 | 3 |
| **WMV** | - | - | - | - | - | - | - | - | - | - | - | - | - | - | - |  | 9 |
|  |  |  |  |  |  |  |  |  |  |  |  |  |  |  |  |  |  |

**Table 5.** Ranked effect sizes (ES) of cooccurrence at Scale 1 (RT-PCR) and Scale 2 (HTS1 & HTS2) associations between the eighteen viruses (*n* = 153 virus-virus combinations).

| **RT-PCR pairs** | **RT-PCR ES** | **HTS1 pairs** | **HTS1 ES** | **HTS2 pairs** | **HTS2 ES** |
| --- | --- | --- | --- | --- | --- |
| PMMoV-RuCMV | 0.033 | BMYV-BChV | 0.191 | PMoV-PZSV | 0.129 |
| RuCMV-TMGMV | 0.028 | PMoV-TuYV | 0.179 | PMoV-TuYV | 0.118 |
| TAV-WMV | 0.028 | PMoV-PZSV | 0.172 | BMYV-TuYV | 0.115 |
| CMV-WMV | 0.026 | BMYV-TuYV | 0.161 | BMYV-BChV | 0.113 |
| BMYV-TuYV | 0.025 | TuYV-BChV | 0.156 | TuYV-BChV | 0.105 |
| TuYV-BChV | 0.023 | TuYV-PZSV | 0.136 | BMYV-PMoV | 0.080 |
| PMMoV-TMV | 0.023 | BMYV-PMoV | 0.107 | PMoV-BChV | 0.077 |
| PMoV-TAV | 0.018 | PMoV-BChV | 0.103 | RuCMV-PZSV | 0.074 |
| RuCMV-TMV | 0.018 | BMYV-PZSV | 0.092 | TuYV-PZSV | 0.074 |
| TMGMV-YoMV | 0.018 | BChV-PZSV | 0.084 | PMoV-TAV | 0.072 |
| CMV-TAV | 0.015 | PMoV-TAV | 0.083 | PMoV-CMV | 0.059 |
| RuCMV-PPV | 0.014 | TMV-WMV | 0.077 | CMV-TAV | 0.055 |
| TAV-TuMV | 0.014 | PMMoV-RuCMV | 0.061 | CMV-PZSV | 0.054 |
| BMYV-BChV | 0.013 | TuYV-TAV | 0.057 | BMYV-PZSV | 0.053 |
| PMMoV-TMGMV | 0.013 | TCV-WMV | 0.054 | TuYV-TAV | 0.053 |
| TMGMV-CMV | 0.012 | CMV-TAV | 0.053 | BChV-PZSV | 0.050 |
| TuYV-TAV | 0.012 | PMMoV-TMGMV | 0.049 | TMGMV-TMV | 0.050 |
| TMGMV-TMV | 0.011 | PZSV-TAV | 0.049 | TMV-CMV | 0.049 |
| CMV-PZSV | 0.010 | TMGMV-TMV | 0.047 | PZSV-TAV | 0.044 |
| PZSV-TAV | 0.010 | PMMoV-TMV | 0.043 | CMV-WMV | 0.044 |
| CABYV-PZSV | 0.009 | CMV-WMV | 0.039 | PMMoV-TMGMV | 0.044 |
| TMV-PPV | 0.009 | RuCMV-WMV | 0.038 | TMV-WMV | 0.042 |
| PMMoV-PZSV | 0.009 | TMGMV-WMV | 0.038 | PMMoV-CMV | 0.037 |
| TMV-CMV | 0.009 | TCV-TMV | 0.037 | PMMoV-RuCMV | 0.037 |
| TCV-CMV | 0.007 | TCV-YoMV | 0.037 | PMMoV-TMV | 0.037 |
| PMoV-PZSV | 0.007 | CABYV-BChV | 0.036 | BMYV-TAV | 0.035 |
| PMMoV-PPV | 0.007 | PMMoV-WMV | 0.035 | BChV-TAV | 0.034 |
| CABYV-RuCMV | 0.006 | PMMoV-PPV | 0.034 | TuYV-CMV | 0.031 |
| BMYV-CABYV | 0.006 | BMYV-CABYV | 0.033 | TAV-WMV | 0.025 |
| PZSV-WMV | 0.006 | TAV-WMV | 0.032 | TCV-CMV | 0.025 |
| BMYV-TAV | 0.005 | CABYV-RuCMV | 0.031 | CABYV-RuCMV | 0.024 |
| PMMoV-WMV | 0.005 | CMV-PZSV | 0.031 | BMYV-CMV | 0.022 |
| PMMoV-CMV | 0.005 | PMMoV-CMV | 0.031 | TCV-WMV | 0.022 |
| RuCMV-PZSV | 0.005 | RuCMV-TMV | 0.030 | TCV-YoMV | 0.022 |
| BYDV-TuYV | 0.003 | TuYV-CMV | 0.030 | CABYV-TuYV | 0.021 |
| PPV-PZSV | 0.003 | BMYV-TAV | 0.029 | PMoV-RuCMV | 0.020 |
| RuCMV-TuMV | 0.003 | CABYV-TuYV | 0.028 | CABYV-BChV | 0.020 |
| CABYV-WMV | 0.003 | RuCMV-PZSV | 0.026 | BMYV-CABYV | 0.019 |
| BChV-TuMV | 0.003 | TMV-YoMV | 0.026 | CMV-BChV | 0.018 |
| CABYV-BChV | 0.003 | WMV-YoMV | 0.026 | PMMoV-PPV | 0.018 |
| PMoV-TMV | 0.003 | CABYV-WMV | 0.025 | CABYV-WMV | 0.016 |
| TuYV-PZSV | 0.003 | RuCMV-PPV | 0.025 | CABYV-PMoV | 0.015 |
| BMYV-PMMoV | 0.002 | BMYV-CMV | 0.024 | TCV-TMV | 0.015 |
| BYDV-BChV | 0.002 | TCV-TuMV | 0.024 | TMGMV-PPV | 0.015 |
| BMYV-PMoV | 0.002 | PMoV-CMV | 0.023 | PMMoV-WMV | 0.014 |
| TCV-TMGMV | 0.002 | TMV-PPV | 0.023 | TCV-TMGMV | 0.014 |
| BMYV-WMV | 0.002 | BChV-TAV | 0.022 | TuMV-WMV | 0.014 |
| TCV-YoMV | 0.002 | TMGMV-PPV | 0.018 | CMV-TuMV | 0.012 |
| TMGMV-PPV | 0.002 | TuMV-WMV | 0.017 | TMV-PPV | 0.011 |
| BMYV-RuCMV | 0.001 | CABYV-PMMoV | 0.014 | TCV-TuMV | 0.011 |
| CMV-YoMV | 0.001 | CABYV-PMoV | 0.014 | TMV-YoMV | 0.011 |
| PMMoV-PMoV | 0.001 | TCV-TMGMV | 0.014 | PMMoV-PZSV | 0.010 |
| PMMoV-TuYV | 0.001 | TMV-CMV | 0.014 | TuYV-TuMV | 0.010 |
| PMoV-TuYV | 0.001 | CABYV-TuMV | 0.013 | BMYV-TuMV | 0.009 |
| BChV-WMV | 0.001 | RuCMV-BChV | 0.012 | TMGMV-YoMV | 0.009 |
| CABYV-PMMoV | 0.001 | BChV-TuMV | 0.011 | RuCMV-CMV | 0.008 |
| BYDV-BMYV | 0.001 | CMV-BChV | 0.011 | TMV-TuMV | 0.008 |
| CABYV-TuYV | 0.001 | CMV-PPV | 0.010 | PMMoV-BChV | 0.008 |
| BYDV-TuMV | 0.001 | PMMoV-TAV | 0.010 | CABYV-TAV | 0.007 |
| CABYV-PMoV | 0.001 | BMYV-RuCMV | 0.008 | CABYV-TMV | 0.007 |
| CABYV-YoMV | 0.001 | CABYV-TAV | 0.008 | TCV-TAV | 0.007 |
| PMMoV-BChV | 0.001 | TMV-TuMV | 0.008 | CABYV-PMMoV | 0.007 |
| TMV-TuMV | 0.001 | BMYV-TuMV | 0.008 | PMMoV-TAV | 0.007 |
| BChV-TAV | 0.000 | CABYV-PZSV | 0.008 | PMMoV-TuYV | 0.007 |
| RuCMV-TCV | 0.000 | TCV-CMV | 0.008 | BChV-TuMV | 0.007 |
| PMMoV-TCV | 0.000 | TuYV-TuMV | 0.008 | TAV-TuMV | 0.007 |
| PMoV-BChV | 0.000 | RuCMV-TMGMV | 0.007 | CABYV-PZSV | 0.006 |
| TuYV-WMV | 0.000 | TAV-TuMV | 0.007 | PPV-PZSV | 0.005 |
| CABYV-TAV | 0.000 | TuMV-YoMV | 0.006 | RuCMV-PPV | 0.005 |
| TCV-TMV | 0.000 | PMMoV-PZSV | 0.005 | BMYV-PMMoV | 0.005 |
| TuYV-TuMV | 0.000 | CMV-TuMV | 0.004 | CABYV-TuMV | 0.005 |
| TMV-PZSV | 0.000 | CABYV-TMGMV | 0.003 | PMMoV-YoMV | 0.004 |
| BChV-PZSV | 0.000 | CABYV-TCV | 0.003 | WMV-YoMV | 0.004 |
| BYDV-TCV | 0.000 | PPV-PZSV | 0.003 | CMV-PPV | 0.004 |
| BYDV-PMoV | 0.000 | CABYV-YoMV | 0.002 | PMoV-TuMV | 0.004 |
| PMoV-TCV | 0.000 | CABYV-TMV | 0.000 | RuCMV-WMV | 0.004 |
| BMYV-TCV | 0.000 | PMMoV-YoMV | 0.000 | TMGMV-TuYV | 0.004 |
| BYDV-CABYV | 0.000 | PMoV-TuMV | 0.000 | PMMoV-PMoV | 0.003 |
| BYDV-PPV | 0.000 | RuCMV-YoMV | -0.001 | TuMV-YoMV | 0.002 |
| CABYV-CMV | 0.000 | TCV-TAV | -0.001 | RuCMV-TMV | 0.002 |
| CABYV-TCV | 0.000 | BMYV-PMMoV | -0.002 | CABYV-YoMV | 0.001 |
| PMoV-WMV | 0.000 | PZSV-TuMV | -0.002 | CMV-YoMV | 0.000 |
| TCV-BChV | 0.000 | PMMoV-BChV | -0.003 | PMMoV-TuMV | 0.000 |
| TCV-PPV | 0.000 | PMoV-RuCMV | -0.003 | TMGMV-BChV | 0.000 |
| PMoV-TuMV | -0.001 | PPV-WMV | -0.003 | TMGMV-CMV | 0.000 |
| TCV-TuYV | -0.001 | RuCMV-TCV | -0.005 | TMGMV-WMV | 0.000 |
| BMYV-PZSV | -0.001 | CABYV-PPV | -0.006 | BMYV-RuCMV | 0.000 |
| CABYV-TuMV | -0.001 | PMMoV-TuYV | -0.006 | CABYV-TCV | 0.000 |
| PMoV-PPV | -0.001 | BYDV-PPV | -0.007 | PPV-WMV | -0.001 |
| TCV-TuMV | -0.001 | PPV-TuMV | -0.008 | PZSV-TuMV | -0.001 |
| BMYV-TuMV | -0.001 | TMGMV-YoMV | -0.008 | TCV-PPV | -0.001 |
| BYDV-TAV | -0.001 | CABYV-CMV | -0.008 | CABYV-CMV | -0.001 |
| CABYV-PPV | -0.001 | TCV-PPV | -0.008 | RuCMV-BChV | -0.001 |
| BMYV-PPV | -0.001 | TMGMV-CMV | -0.008 | CABYV-PPV | -0.002 |
| BYDV-PMMoV | -0.001 | PPV-YoMV | -0.009 | CABYV-TMGMV | -0.002 |
| BChV-PPV | -0.001 | PMMoV-TuMV | -0.010 | RuCMV-TuYV | -0.003 |
| BYDV-TMV | -0.001 | RuCMV-TuYV | -0.010 | RuCMV-YoMV | -0.003 |
| BYDV-WMV | -0.001 | TMGMV-TuMV | -0.011 | BMYV-PPV | -0.003 |
| BYDV-YoMV | -0.001 | BMYV-PPV | -0.012 | PPV-TuMV | -0.003 |
| TCV-WMV | -0.001 | BYDV-CABYV | -0.012 | PPV-YoMV | -0.004 |
| PMMoV-TuMV | -0.001 | BYDV-TuMV | -0.015 | PMMoV-TCV | -0.004 |
| TuMV-WMV | -0.001 | BYDV-TCV | -0.017 | TMV-TAV | -0.004 |
| BYDV-PZSV | -0.002 | BYDV-YoMV | -0.018 | TMGMV-TuMV | -0.005 |
| TCV-PZSV | -0.002 | BChV-PPV | -0.019 | BChV-PPV | -0.006 |
| CMV-PPV | -0.002 | TMV-TAV | -0.019 | TMGMV-TAV | -0.006 |
| TuYV-PPV | -0.002 | TuYV-PPV | -0.019 | TuYV-PPV | -0.006 |
| PPV-TuMV | -0.002 | BYDV-BChV | -0.020 | RuCMV-TCV | -0.007 |
| PPV-YoMV | -0.002 | PMMoV-PMoV | -0.022 | PMoV-PPV | -0.007 |
| RuCMV-BChV | -0.002 | BYDV-BMYV | -0.023 | PPV-TAV | -0.008 |
| BMYV-YoMV | -0.002 | PMoV-PPV | -0.023 | BChV-YoMV | -0.008 |
| BYDV-RuCMV | -0.002 | RuCMV-TuMV | -0.023 | TAV-YoMV | -0.009 |
| BYDV-TMGMV | -0.002 | CMV-YoMV | -0.025 | BMYV-TMGMV | -0.009 |
| TCV-TAV | -0.002 | PPV-TAV | -0.025 | BMYV-YoMV | -0.009 |
| BYDV-CMV | -0.003 | BChV-WMV | -0.027 | BYDV-PPV | -0.011 |
| PMoV-YoMV | -0.003 | BMYV-WMV | -0.027 | PZSV-YoMV | -0.011 |
| CABYV-TMV | -0.003 | TMGMV-BChV | -0.029 | BChV-WMV | -0.013 |
| BMYV-TMV | -0.004 | BChV-YoMV | -0.031 | BMYV-WMV | -0.013 |
| PPV-WMV | -0.004 | PMMoV-TCV | -0.031 | TuYV-YoMV | -0.013 |
| BChV-YoMV | -0.004 | BYDV-PMMoV | -0.033 | RuCMV-TuMV | -0.013 |
| TMV-BChV | -0.004 | BMYV-TMGMV | -0.034 | TMV-BChV | -0.014 |
| WMV-YoMV | -0.005 | RuCMV-CMV | -0.034 | BMYV-TMV | -0.015 |
| RuCMV-TuYV | -0.005 | TMGMV-TAV | -0.034 | BMYV-TCV | -0.015 |
| TMGMV-TuMV | -0.005 | BMYV-YoMV | -0.035 | PMoV-WMV | -0.015 |
| TuMV-YoMV | -0.005 | BYDV-TMV | -0.039 | PMoV-YoMV | -0.016 |
| PMMoV-YoMV | -0.005 | BYDV-TuYV | -0.039 | TCV-TuYV | -0.016 |
| PZSV-TuMV | -0.005 | BYDV-WMV | -0.039 | BYDV-CABYV | -0.017 |
| PMoV-RuCMV | -0.006 | BMYV-TCV | -0.041 | RuCMV-TAV | -0.017 |
| TMGMV-BChV | -0.006 | BYDV-TAV | -0.041 | TCV-BChV | -0.017 |
| RuCMV-YoMV | -0.006 | TMGMV-TuYV | -0.041 | TuYV-WMV | -0.019 |
| TMV-TuYV | -0.006 | TCV-TuYV | -0.043 | PMoV-TCV | -0.020 |
| TuYV-YoMV | -0.006 | BMYV-TMV | -0.044 | BYDV-TuMV | -0.022 |
| TMV-YoMV | -0.007 | TMV-BChV | -0.044 | TMGMV-PZSV | -0.023 |
| PPV-TAV | -0.007 | TCV-BChV | -0.045 | TMV-PZSV | -0.023 |
| BMYV-TMGMV | -0.007 | PZSV-WMV | -0.046 | TMV-TuYV | -0.023 |
| PMoV-CMV | -0.007 | TAV-YoMV | -0.047 | PZSV-WMV | -0.025 |
| RuCMV-WMV | -0.009 | TuYV-YoMV | -0.047 | BYDV-YoMV | -0.026 |
| BMYV-CMV | -0.009 | PMoV-TCV | -0.052 | RuCMV-TMGMV | -0.026 |
| PMMoV-TAV | -0.009 | TMGMV-PZSV | -0.052 | PMoV-TMV | -0.027 |
| TMGMV-WMV | -0.009 | PZSV-YoMV | -0.053 | BYDV-TCV | -0.030 |
| CABYV-TMGMV | -0.010 | BYDV-RuCMV | -0.053 | PMoV-TMGMV | -0.032 |
| PMoV-TMGMV | -0.010 | BYDV-PMoV | -0.055 | BYDV-BChV | -0.036 |
| CMV-TuMV | -0.010 | TuYV-WMV | -0.055 | BYDV-BMYV | -0.040 |
| CMV-BChV | -0.011 | PMoV-YoMV | -0.056 | TCV-PZSV | -0.042 |
| TAV-YoMV | -0.011 | BYDV-TMGMV | -0.057 | BYDV-TAV | -0.051 |
| TMGMV-TuYV | -0.011 | RuCMV-TAV | -0.059 | BYDV-PMMoV | -0.052 |
| TMV-WMV | -0.012 | PMoV-WMV | -0.066 | BYDV-TuYV | -0.059 |
| RuCMV-CMV | -0.015 | TMV-PZSV | -0.071 | BYDV-PMoV | -0.072 |
| TuYV-CMV | -0.015 | BYDV-PZSV | -0.072 | BYDV-WMV | -0.074 |
| PZSV-YoMV | -0.016 | TCV-PZSV | -0.072 | BYDV-TMV | -0.079 |
| TMV-TAV | -0.016 | TMV-TuYV | -0.081 | BYDV-RuCMV | -0.115 |
| TMGMV-PZSV | -0.024 | BYDV-CMV | -0.081 | BYDV-TMGMV | -0.116 |
| RuCMV-TAV | -0.028 | PMoV-TMGMV | -0.082 | BYDV-PZSV | -0.139 |
| TMGMV-TAV | -0.035 | PMoV-TMV | -0.108 | BYDV-CMV | -0.170 |
|  |  |  |  |  |  |

**Table 6.** Cooccurrence analyses showing probabilities (α = 0.05) that the two viruses cooccur at Scale 1 (RT-PCR) or Scale 2 (HTS1 & HTS2) at a frequency less, *P(lt)* or more, *P(gt)*, than the observed number of two species that were distributed randomly (independently) of one another. *P*(*lt*) <= α suggests that those two species are negatively (highlighted with red cells) associated and positively with green (where *P*(*gt*) <= α; the pair co-occurs at a rate higher than we would expect to find by chance).

| **Virus pair** | **RT-PCR *P*(*lt*)** | **HTS1 *P*(*lt*)** | **HTS2 *P*(*lt*)** | **RT-PCR *P*(*gt*)** | **HTS1 *P*(*gt*)** | **HTS2 *P*(*gt*)** |
| --- | --- | --- | --- | --- | --- | --- |
| BChV-PPV | 0.408 | 0.066 | 0.128 | 1.000 | 1.000 | 1.000 |
| BChV-PZSV | 0.607 | 1.000 | 1.000 | 0.608 | 0.000 | 0.000 |
| BChV-TAV | 0.659 | 0.896 | 1.000 | 0.525 | 0.195 | 0.000 |
| BChV-TuMV | 0.964 | 0.857 | 0.929 | 0.118 | 0.310 | 0.171 |
| BChV-WMV | 0.800 | 0.124 | 0.115 | 0.392 | 0.944 | 0.943 |
| BChV-YoMV | 0.038 | 0.035 | 0.139 | 1.000 | 0.993 | 0.959 |
| BMYV-BChV | 1.000 | 1.000 | 1.000 | 0.000 | 0.000 | 0.000 |
| BMYV-CABYV | 1.000 | 0.997 | 1.000 | 0.001 | 0.016 | 0.001 |
| BMYV-CMV | 0.002 | 0.960 | 0.993 | 1.000 | 0.117 | 0.017 |
| BMYV-PMMoV | 0.925 | 0.558 | 0.790 | 0.199 | 0.613 | 0.331 |
| BMYV-PMoV | 0.974 | 1.000 | 1.000 | 0.141 | 0.000 | 0.000 |
| BMYV-PPV | 0.451 | 0.245 | 0.373 | 1.000 | 0.950 | 0.894 |
| BMYV-PZSV | 0.492 | 1.000 | 1.000 | 0.734 | 0.000 | 0.000 |
| BMYV-RuCMV | 0.791 | 0.726 | 0.544 | 0.373 | 0.417 | 0.577 |
| BMYV-TAV | 0.983 | 0.937 | 1.000 | 0.048 | 0.127 | 0.000 |
| BMYV-TCV | 0.761 | 0.005 | 0.012 | 1.000 | 1.000 | 0.999 |
| BMYV-TMGMV | 0.014 | 0.084 | 0.240 | 0.997 | 0.962 | 0.844 |
| BMYV-TMV | 0.058 | 0.024 | 0.085 | 1.000 | 0.992 | 0.958 |
| BMYV-TuMV | 0.479 | 0.798 | 0.961 | 0.835 | 0.396 | 0.103 |
| BMYV-TuYV | 1.000 | 1.000 | 1.000 | 0.000 | 0.000 | 0.000 |
| BMYV-WMV | 0.861 | 0.130 | 0.118 | 0.306 | 0.940 | 0.939 |
| BMYV-YoMV | 0.230 | 0.021 | 0.102 | 0.944 | 0.997 | 0.972 |
| BYDV-BChV | 0.998 | 0.120 | 0.000 | 0.033 | 0.970 | 1.000 |
| BYDV-BMYV | 0.973 | 0.084 | 0.000 | 0.239 | 0.981 | 1.000 |
| BYDV-CABYV | 0.769 | 0.203 | 0.002 | 1.000 | 1.000 | 1.000 |
| BYDV-CMV | 0.150 | 0.000 | 0.000 | 0.973 | 1.000 | 1.000 |
| BYDV-PMMoV | 0.438 | 0.007 | 0.000 | 1.000 | 1.000 | 1.000 |
| BYDV-PMoV | 0.797 | 0.000 | 0.000 | 1.000 | 1.000 | 1.000 |
| BYDV-PPV | 0.769 | 0.403 | 0.020 | 1.000 | 1.000 | 1.000 |
| BYDV-PZSV | 0.265 | 0.000 | 0.000 | 1.000 | 1.000 | 1.000 |
| BYDV-RuCMV | 0.156 | 0.000 | 0.000 | 1.000 | 1.000 | 1.000 |
| BYDV-TAV | 0.468 | 0.004 | 0.000 | 0.853 | 1.000 | 1.000 |
| BYDV-TCV | 0.914 | 0.098 | 0.000 | 1.000 | 1.000 | 1.000 |
| BYDV-TMGMV | 0.178 | 0.000 | 0.000 | 0.966 | 1.000 | 1.000 |
| BYDV-TMV | 0.391 | 0.002 | 0.000 | 1.000 | 1.000 | 1.000 |
| BYDV-TuMV | 0.895 | 0.132 | 0.000 | 0.448 | 1.000 | 1.000 |
| BYDV-TuYV | 0.999 | 0.007 | 0.000 | 0.008 | 0.999 | 1.000 |
| BYDV-WMV | 0.367 | 0.002 | 0.000 | 1.000 | 1.000 | 1.000 |
| BYDV-YoMV | 0.386 | 0.085 | 0.000 | 1.000 | 1.000 | 1.000 |
| CABYV-BChV | 0.991 | 0.999 | 1.000 | 0.051 | 0.009 | 0.001 |
| CABYV-CMV | 0.536 | 0.330 | 0.502 | 0.638 | 0.880 | 0.686 |
| CABYV-PMMoV | 0.821 | 0.923 | 0.939 | 0.393 | 0.214 | 0.156 |
| CABYV-PMoV | 0.868 | 0.901 | 0.996 | 0.484 | 0.254 | 0.015 |
| CABYV-PPV | 0.467 | 0.462 | 0.462 | 1.000 | 1.000 | 1.000 |
| CABYV-PZSV | 1.000 | 0.811 | 0.878 | 0.001 | 0.419 | 0.247 |
| CABYV-RuCMV | 0.990 | 0.995 | 1.000 | 0.031 | 0.028 | 0.000 |
| CABYV-TAV | 0.642 | 0.826 | 0.943 | 0.559 | 0.370 | 0.147 |
| CABYV-TCV | 0.769 | 0.763 | 0.653 | 1.000 | 0.544 | 0.641 |
| CABYV-TMGMV | 0.001 | 0.704 | 0.464 | 1.000 | 0.530 | 0.714 |
| CABYV-TMV | 0.065 | 0.644 | 0.923 | 1.000 | 0.607 | 0.175 |
| CABYV-TuMV | 0.503 | 0.954 | 0.944 | 0.822 | 0.180 | 0.189 |
| CABYV-TuYV | 0.861 | 0.991 | 1.000 | 0.383 | 0.043 | 0.001 |
| CABYV-WMV | 0.958 | 0.987 | 0.997 | 0.120 | 0.054 | 0.012 |
| CABYV-YoMV | 0.751 | 0.732 | 0.743 | 0.484 | 0.578 | 0.549 |
| CMV-BChV | 0.001 | 0.825 | 0.980 | 1.000 | 0.349 | 0.044 |
| CMV-PPV | 0.362 | 1.000 | 0.846 | 0.789 | 0.262 | 0.356 |
| CMV-PZSV | 0.957 | 0.981 | 1.000 | 0.067 | 0.057 | 0.000 |
| CMV-TAV | 0.984 | 1.000 | 1.000 | 0.025 | 0.001 | 0.000 |
| CMV-TuMV | 0.015 | 0.769 | 0.970 | 0.994 | 0.513 | 0.081 |
| CMV-WMV | 1.000 | 0.998 | 1.000 | 0.000 | 0.012 | 0.000 |
| CMV-YoMV | 0.646 | 0.054 | 0.576 | 0.448 | 0.986 | 0.586 |
| PMMoV-BChV | 0.735 | 0.532 | 0.871 | 0.501 | 0.642 | 0.224 |
| PMMoV-CMV | 0.859 | 0.992 | 1.000 | 0.206 | 0.039 | 0.000 |
| PMMoV-PMoV | 0.878 | 0.192 | 0.687 | 0.309 | 0.900 | 0.431 |
| PMMoV-PPV | 0.999 | 1.000 | 1.000 | 0.004 | 0.002 | 0.000 |
| PMMoV-PZSV | 0.989 | 0.686 | 0.854 | 0.024 | 0.482 | 0.222 |
| PMMoV-RuCMV | 1.000 | 0.999 | 1.000 | 0.000 | 0.003 | 0.001 |
| PMMoV-TAV | 0.021 | 0.846 | 0.888 | 0.992 | 0.323 | 0.216 |
| PMMoV-TCV | 0.821 | 0.090 | 0.409 | 0.562 | 0.961 | 0.723 |
| PMMoV-TMGMV | 0.993 | 0.996 | 1.000 | 0.013 | 0.013 | 0.000 |
| PMMoV-TMV | 1.000 | 0.992 | 1.000 | 0.000 | 0.024 | 0.001 |
| PMMoV-TuMV | 0.429 | 0.346 | 0.618 | 0.756 | 0.853 | 0.586 |
| PMMoV-TuYV | 0.798 | 0.474 | 0.817 | 0.371 | 0.683 | 0.282 |
| PMMoV-WMV | 0.948 | 0.976 | 0.940 | 0.102 | 0.060 | 0.107 |
| PMMoV-YoMV | 0.086 | 0.615 | 0.800 | 0.967 | 0.609 | 0.353 |
| PMoV-BChV | 0.829 | 1.000 | 1.000 | 0.541 | 0.000 | 0.000 |
| PMoV-CMV | 0.008 | 0.941 | 1.000 | 0.999 | 0.142 | 0.000 |
| PMoV-PPV | 0.516 | 0.038 | 0.113 | 1.000 | 0.996 | 0.980 |
| PMoV-PZSV | 0.999 | 1.000 | 1.000 | 0.006 | 0.000 | 0.000 |
| PMoV-RuCMV | 0.009 | 0.530 | 0.956 | 1.000 | 0.616 | 0.074 |
| PMoV-TAV | 1.000 | 1.000 | 1.000 | 0.000 | 0.000 | 0.000 |
| PMoV-TCV | 0.797 | 0.001 | 0.005 | 1.000 | 1.000 | 0.999 |
| PMoV-TMGMV | 0.000 | 0.000 | 0.008 | 1.000 | 1.000 | 0.996 |
| PMoV-TMV | 0.941 | 0.000 | 0.012 | 0.169 | 1.000 | 0.995 |
| PMoV-TuMV | 0.577 | 0.605 | 0.783 | 0.776 | 0.612 | 0.371 |
| PMoV-TuYV | 0.900 | 1.000 | 1.000 | 0.319 | 0.000 | 0.000 |
| PMoV-WMV | 0.578 | 0.002 | 0.109 | 0.702 | 0.999 | 0.937 |
| PMoV-YoMV | 0.091 | 0.001 | 0.020 | 1.000 | 1.000 | 0.995 |
| PPV-PZSV | 0.954 | 0.723 | 0.899 | 0.121 | 0.597 | 0.250 |
| PPV-TAV | 0.004 | 0.020 | 0.068 | 1.000 | 1.000 | 1.000 |
| PPV-TuMV | 0.178 | 0.376 | 0.358 | 1.000 | 1.000 | 1.000 |
| PPV-WMV | 0.054 | 0.577 | 0.594 | 1.000 | 0.739 | 0.667 |
| PPV-YoMV | 0.251 | 0.304 | 0.301 | 0.937 | 1.000 | 1.000 |
| PZSV-TAV | 0.967 | 0.995 | 1.000 | 0.054 | 0.016 | 0.000 |
| PZSV-TuMV | 0.087 | 0.567 | 0.544 | 0.967 | 0.662 | 0.620 |
| PZSV-WMV | 0.933 | 0.020 | 0.028 | 0.116 | 0.993 | 0.985 |
| PZSV-YoMV | 0.000 | 0.001 | 0.133 | 1.000 | 1.000 | 0.933 |
| RuCMV-BChV | 0.314 | 0.774 | 0.518 | 0.843 | 0.362 | 0.606 |
| RuCMV-CMV | 0.024 | 0.042 | 0.774 | 0.985 | 0.987 | 0.305 |
| RuCMV-PPV | 1.000 | 0.997 | 0.899 | 0.000 | 0.031 | 0.245 |
| RuCMV-PZSV | 0.848 | 0.922 | 1.000 | 0.218 | 0.155 | 0.000 |
| RuCMV-TAV | 0.000 | 0.007 | 0.090 | 1.000 | 0.998 | 0.949 |
| RuCMV-TCV | 0.784 | 0.474 | 0.276 | 0.516 | 0.723 | 0.832 |
| RuCMV-TMGMV | 1.000 | 0.686 | 0.038 | 0.000 | 0.455 | 0.977 |
| RuCMV-TMV | 1.000 | 0.941 | 0.609 | 0.000 | 0.120 | 0.489 |
| RuCMV-TuMV | 0.857 | 0.106 | 0.062 | 0.235 | 0.967 | 0.976 |
| RuCMV-TuYV | 0.116 | 0.405 | 0.454 | 0.950 | 0.730 | 0.650 |
| RuCMV-WMV | 0.042 | 0.975 | 0.656 | 0.979 | 0.059 | 0.443 |
| RuCMV-YoMV | 0.118 | 0.587 | 0.439 | 0.933 | 0.616 | 0.708 |
| TAV-TuMV | 1.000 | 0.763 | 0.908 | 0.001 | 0.435 | 0.199 |
| TAV-WMV | 1.000 | 0.956 | 0.995 | 0.000 | 0.095 | 0.012 |
| TAV-YoMV | 0.012 | 0.003 | 0.137 | 0.995 | 1.000 | 0.952 |
| TCV-BChV | 0.734 | 0.001 | 0.002 | 1.000 | 1.000 | 1.000 |
| TCV-CMV | 1.000 | 0.832 | 1.000 | 0.000 | 0.417 | 0.002 |
| TCV-PPV | 0.769 | 0.326 | 0.613 | 1.000 | 1.000 | 0.759 |
| TCV-PZSV | 0.265 | 0.000 | 0.000 | 1.000 | 1.000 | 1.000 |
| TCV-TAV | 0.147 | 0.596 | 0.897 | 1.000 | 0.613 | 0.201 |
| TCV-TMGMV | 0.903 | 0.870 | 0.964 | 0.278 | 0.277 | 0.076 |
| TCV-TMV | 0.783 | 0.996 | 0.980 | 0.609 | 0.017 | 0.047 |
| TCV-TuMV | 0.553 | 0.992 | 0.988 | 1.000 | 0.041 | 0.042 |
| TCV-TuYV | 0.623 | 0.007 | 0.018 | 1.000 | 0.999 | 0.996 |
| TCV-WMV | 0.367 | 1.000 | 0.998 | 1.000 | 0.001 | 0.006 |
| TCV-YoMV | 0.950 | 0.999 | 1.000 | 0.221 | 0.005 | 0.000 |
| TMGMV-BChV | 0.055 | 0.125 | 0.565 | 0.980 | 0.939 | 0.560 |
| TMGMV-CMV | 0.936 | 0.408 | 0.549 | 0.088 | 0.767 | 0.547 |
| TMGMV-PPV | 0.767 | 0.987 | 0.999 | 0.385 | 0.096 | 0.008 |
| TMGMV-PZSV | 0.000 | 0.013 | 0.065 | 1.000 | 0.996 | 0.959 |
| TMGMV-TAV | 0.000 | 0.095 | 0.341 | 1.000 | 0.954 | 0.758 |
| TMGMV-TMV | 0.981 | 0.992 | 1.000 | 0.033 | 0.023 | 0.000 |
| TMGMV-TuMV | 0.180 | 0.334 | 0.347 | 0.891 | 0.836 | 0.792 |
| TMGMV-TuYV | 0.003 | 0.053 | 0.666 | 0.999 | 0.976 | 0.435 |
| TMGMV-WMV | 0.059 | 0.977 | 0.550 | 0.965 | 0.056 | 0.553 |
| TMGMV-YoMV | 0.999 | 0.413 | 0.896 | 0.002 | 0.767 | 0.193 |
| TMV-BChV | 0.040 | 0.021 | 0.088 | 1.000 | 0.994 | 0.957 |
| TMV-CMV | 0.954 | 0.866 | 1.000 | 0.074 | 0.286 | 0.000 |
| TMV-PPV | 1.000 | 0.995 | 0.992 | 0.000 | 0.039 | 0.036 |
| TMV-PZSV | 0.576 | 0.001 | 0.048 | 0.555 | 1.000 | 0.972 |
| TMV-TAV | 0.000 | 0.251 | 0.406 | 1.000 | 0.858 | 0.709 |
| TMV-TuMV | 0.674 | 0.819 | 0.911 | 0.501 | 0.367 | 0.180 |
| TMV-TuYV | 0.007 | 0.000 | 0.024 | 1.000 | 1.000 | 0.989 |
| TMV-WMV | 0.000 | 1.000 | 1.000 | 1.000 | 0.000 | 0.000 |
| TMV-YoMV | 0.043 | 0.971 | 0.944 | 0.986 | 0.085 | 0.118 |
| TuMV-WMV | 0.434 | 0.930 | 0.985 | 0.738 | 0.181 | 0.041 |
| TuMV-YoMV | 0.059 | 0.828 | 0.805 | 0.985 | 0.409 | 0.421 |
| TuYV-BChV | 1.000 | 1.000 | 1.000 | 0.000 | 0.000 | 0.000 |
| TuYV-CMV | 0.000 | 0.977 | 0.998 | 1.000 | 0.070 | 0.005 |
| TuYV-PPV | 0.253 | 0.075 | 0.175 | 1.000 | 0.991 | 0.965 |
| TuYV-PZSV | 0.846 | 1.000 | 1.000 | 0.275 | 0.000 | 0.000 |
| TuYV-TAV | 0.999 | 0.997 | 1.000 | 0.002 | 0.010 | 0.000 |
| TuYV-TuMV | 0.668 | 0.778 | 0.953 | 0.572 | 0.415 | 0.110 |
| TuYV-WMV | 0.655 | 0.009 | 0.052 | 0.537 | 0.997 | 0.974 |
| TuYV-YoMV | 0.007 | 0.004 | 0.051 | 1.000 | 0.999 | 0.985 |
| WMV-YoMV | 0.143 | 0.971 | 0.781 | 0.930 | 0.085 | 0.364 |
|  |  |  |  |  |  |  |

**Table 7:** Summary of ecological strategies based on the analyses presented in Table S6. Significantly positive (green cells) and negative (red cells) for analyses of the RT-PCR and HTS datasets, and the number of virus-virus combinations are given for each mode of cooccurrence and ‘ecological strategy’ that groups the cooccurrence categories.

| Scale 1 | Scale 2 | |  |  |  |  |
| --- | --- | --- | --- | --- | --- | --- |
| **RT-PCR** | **HTS1** | **HTS2** | **Cooccurrence** | ***n*** | **Ecological strategy** | ***n*** |
| + |  |  | Scale 1 | 5 | uniform scale-specific | 66 |
| - |  |  | Scale 1 | 10 |  |  |
|  | + | + | Scale 2 | 22 |  |  |
|  |  | + |  |  |  |  |
|  | - | - | Scale 2 | 29 |  |  |
|  |  | - |  |  |  |  |
|  | - |  |  |  |  |  |
| + | + | + | Scale 1-Scale 2 | 21 | uniform across-scale | 32 |
| + | + |  |  |  |  |  |
| + |  | + |  |  |  |  |
| - | - | - | Scale 1-Scale 2 | 11 |  |  |
| - | - |  |  |  |  |  |
| - | + | + | Scale 1-Scale 2 | 5 | contrasting across-scale | 8 |
| - |  | + |  |  |  |  |
| + | - | - | Scale 1-Scale 2 | 3 |  |  |
| + |  | - |  |  |  |  |
|  |  |  | no pattern |  | no pattern | 47 |

**
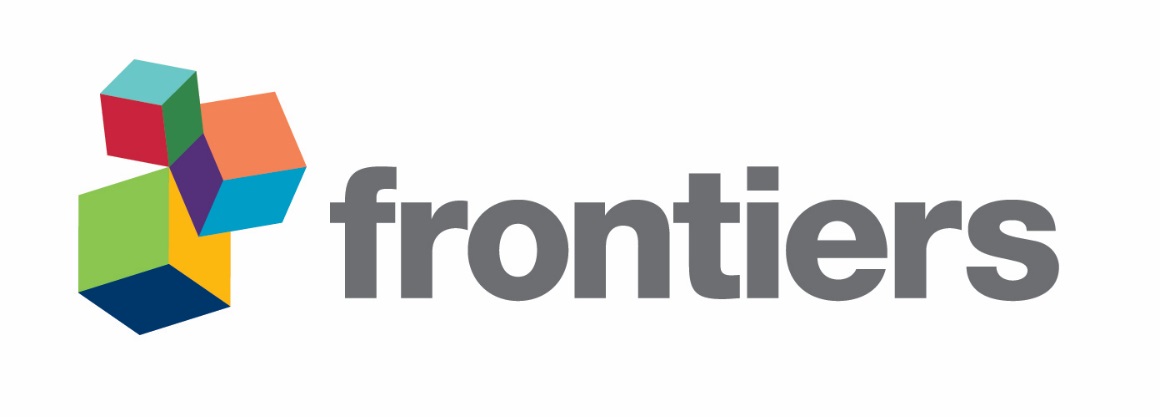
**


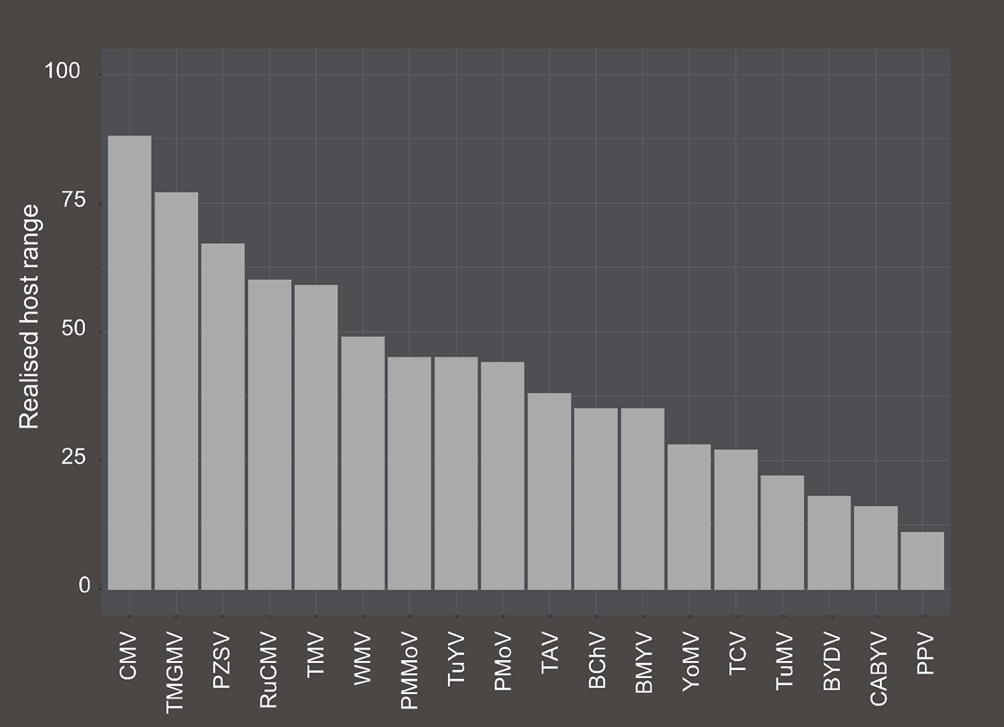


**Supplementary Figure 1.** Host range estimates based on the HTS detections for the eighteen viruses detected with RT-PCR from individual plant samples.

**
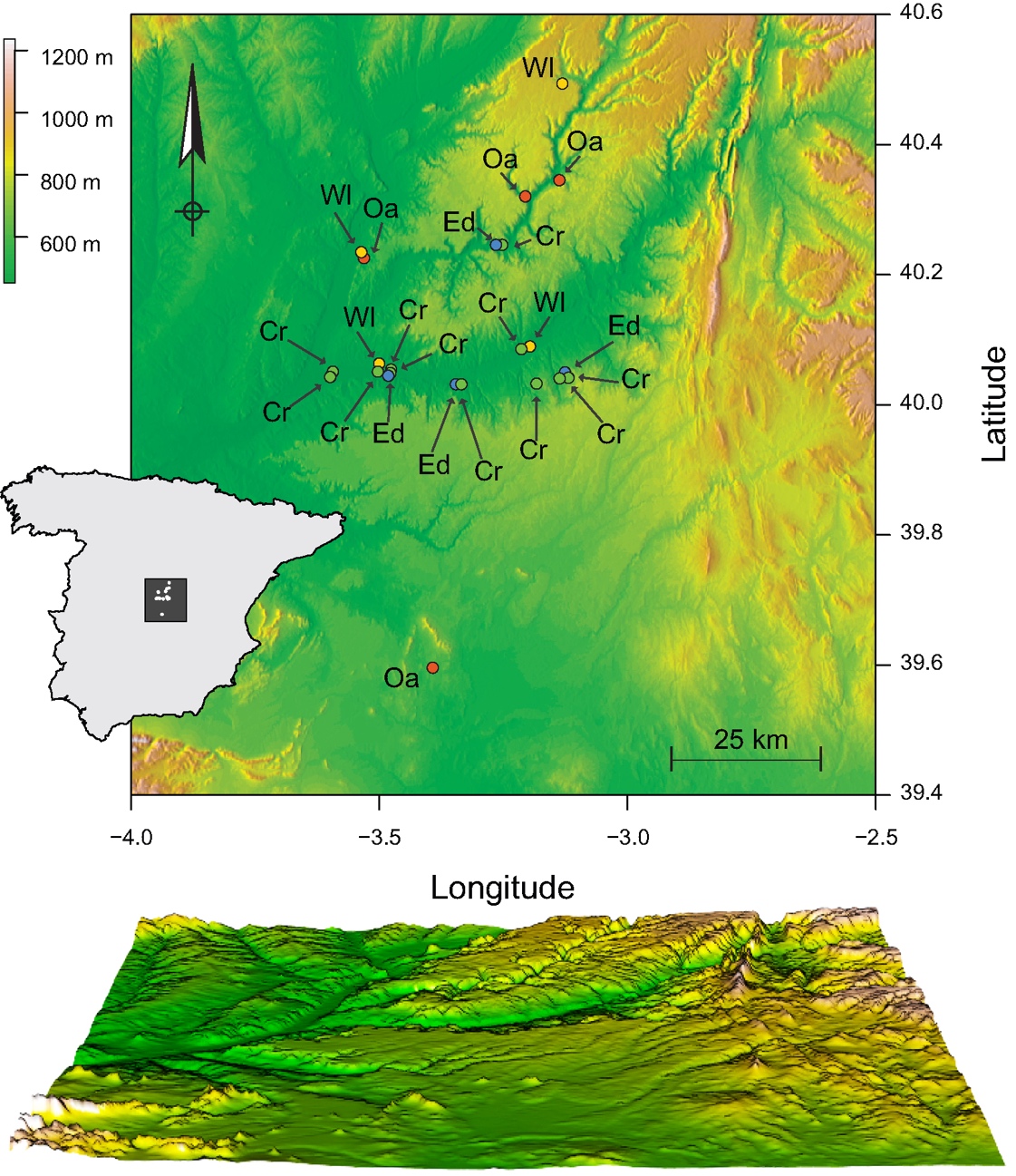
**

**Supplementary Figure 2:** Locations of study sites that comprise four categories of habitat: Crop (Cr = green sites), Edge (Ed = blue sites), Oak (Ok = red sites), and Wasteland (Wl = yellow sites). Inset of the Iberian Peninsula shows location of the extent of the study. The lower panel is a relief map of the study area.


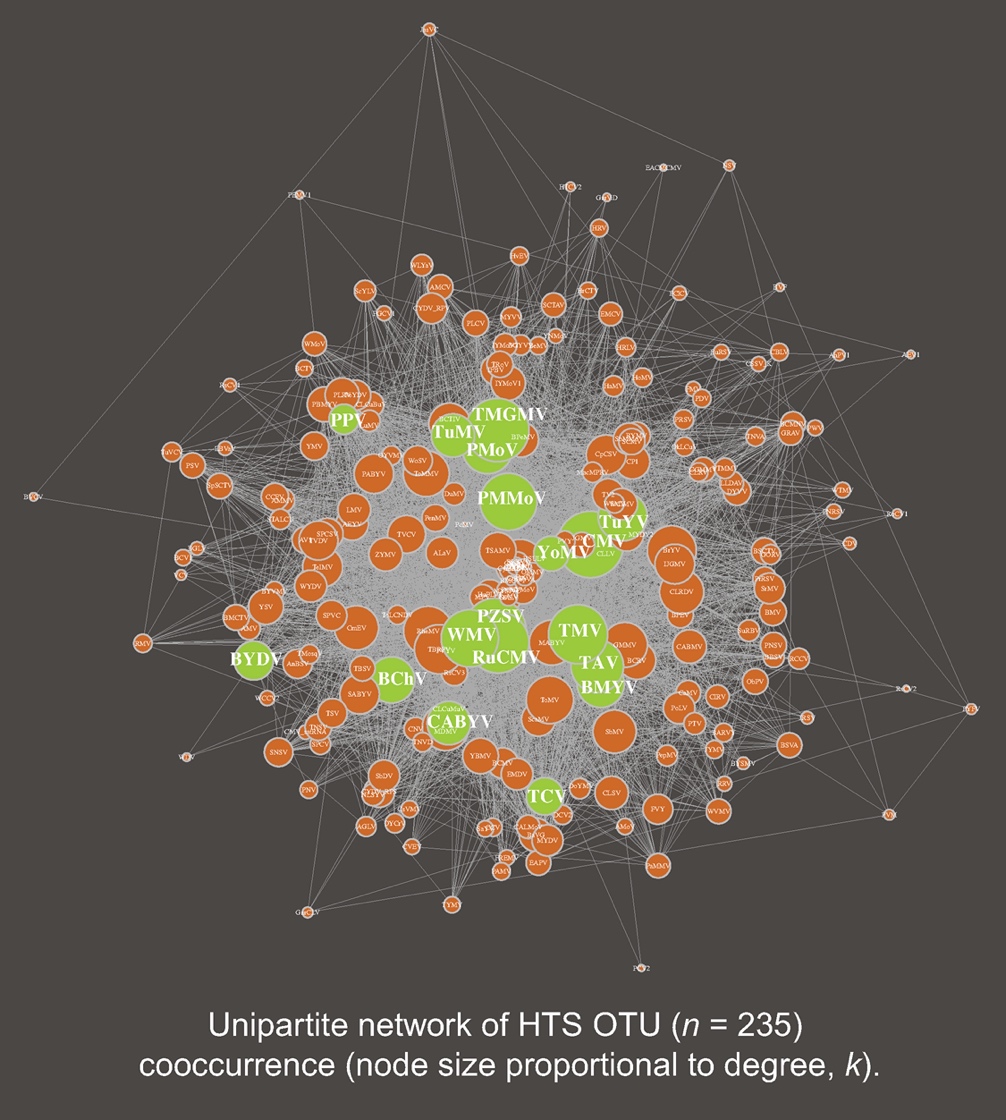


**Supplementary Figure 3.** Unipartite network of cooccurrences of HTS OTUs in host species (*n* = 310 HTS libraries, *n* = 113 host species). Each link in the network (i.e., a link between two nodes) represents an instance of cooccurrence between OTUs. There were 4,360 cooccurrence OTU-OTU pairs. The eighteen viruses of interest used are highlighted with green nodes. The node size is proportional to the number of other nodes it is connected with. Viruses that did not cooccur with any other were omitted from the network (*n* = 5).


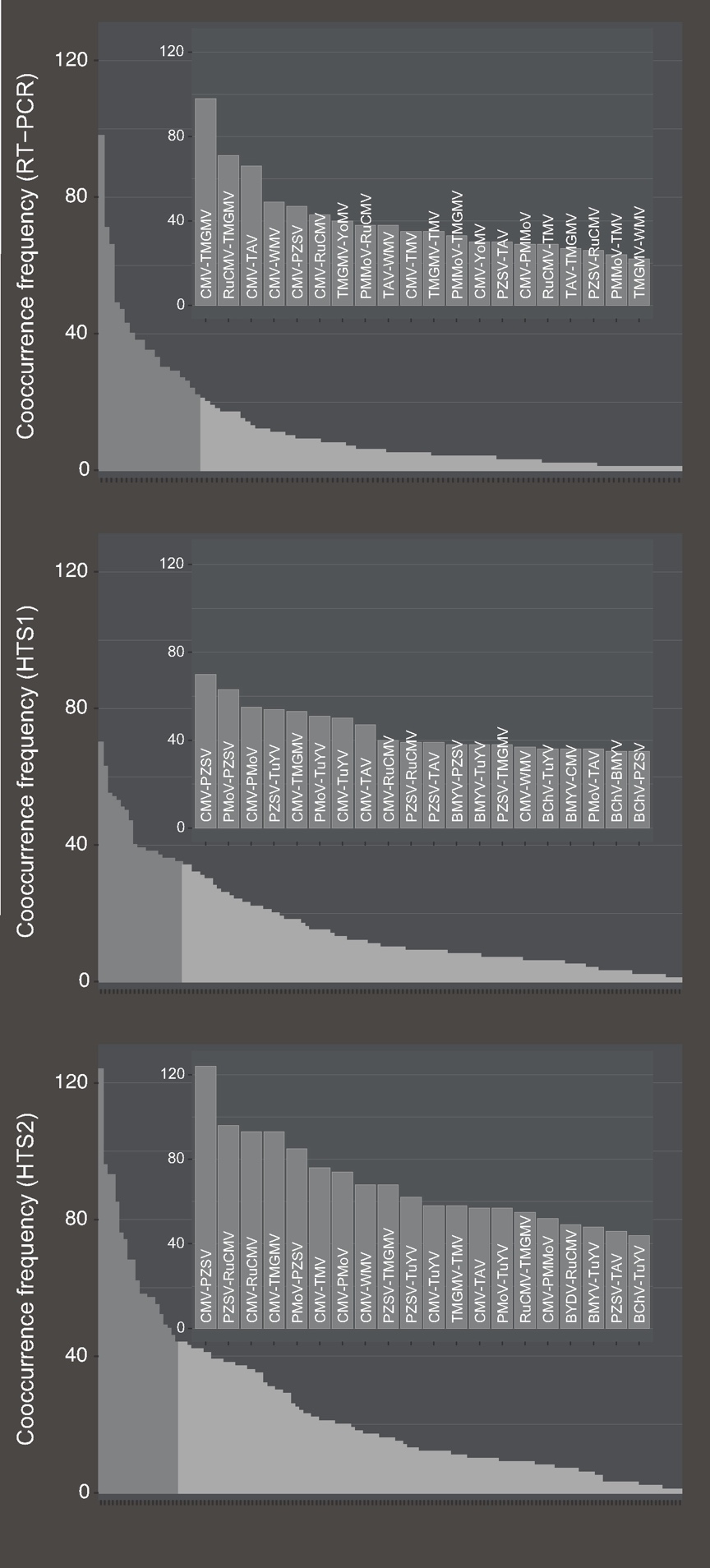


**Supplementary Figure 4.** Distributions of cooccurences at Scale 1 (RT-PCR) and Scale 2 (HTS1 and HTS2) pairs. Top 20 frequencies of each distribution shown in insets.

**
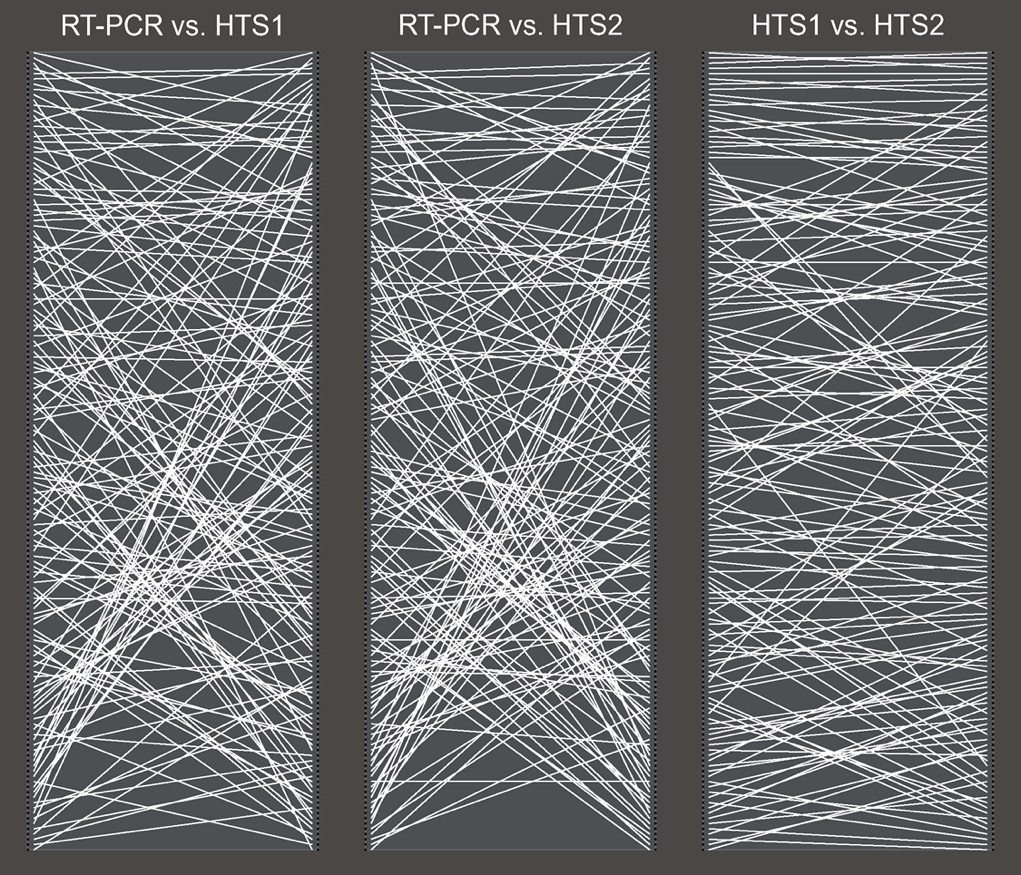
**

**Supplementary Figure 5.** Graphic representation of connectivity among ranked cooccurrences (between RT-PCR and HTS and between HTS datasets) effect sizes of all 153 virus pair combinations. The greatest disturbances to virus pair ranks occurred between the RT-PCR and either of the HTS estimates of effect size, which indicated a change in pattern between the two scales of organisation.

**
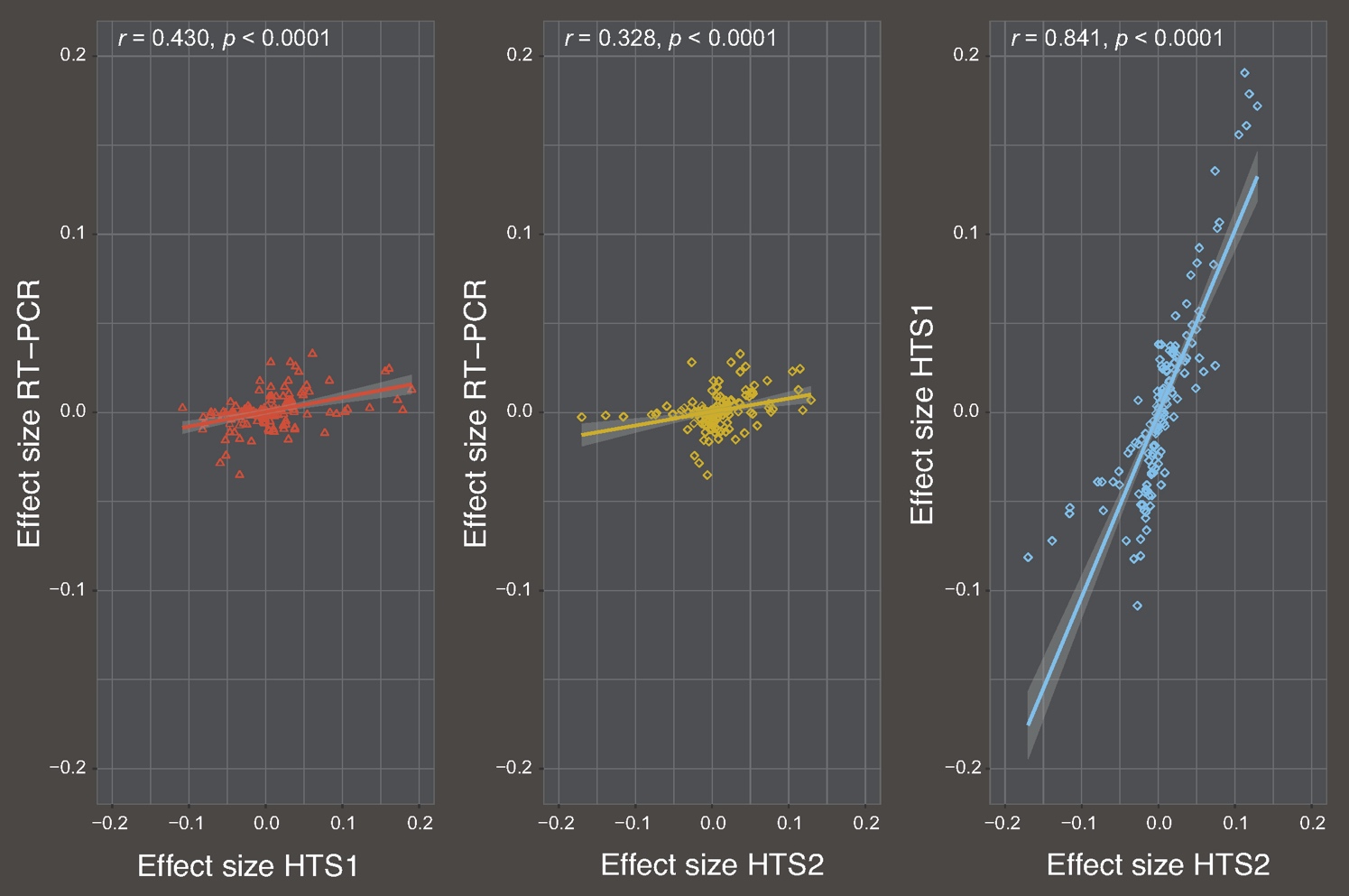
**

**Supplementary Figure 6.** Pearson’s correlations between the effect sizes derived from RT-PCR, HTS1, and HTS2 datasets. The virus pair cooccurrences of the HTS datasets shared relatively more negative and positive effects size similarities (right panel) compared to those between the RT-PCR and either of the HTS data (left and centre panels). The correlations (*r*) statistic and *p*-values are given at the top of each panel.


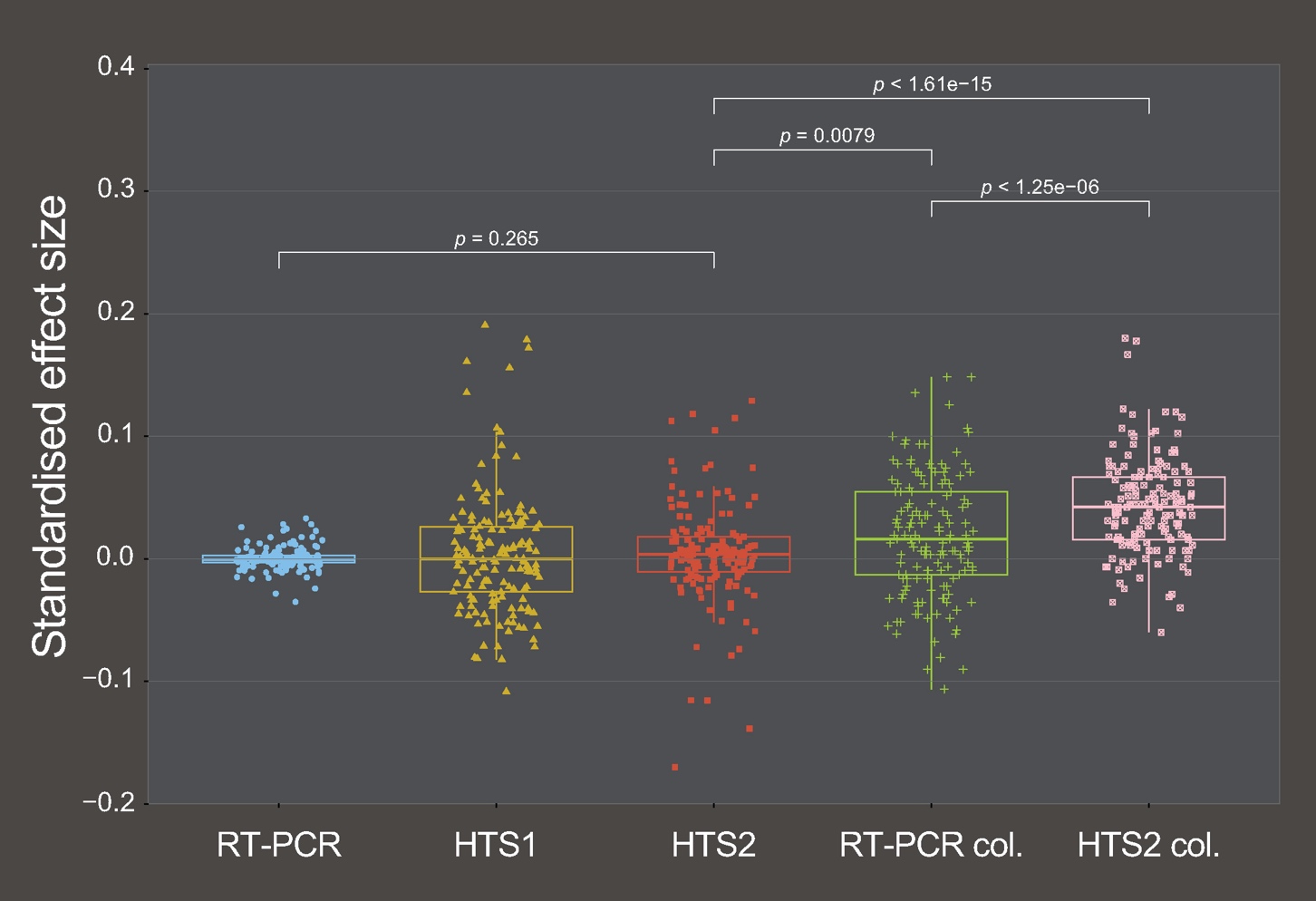


**Supplementary Figure 7.** Student *t*-tests between effect sizes based on RT-PCR samples, HTS libraries, and the same data aggregated by collection. *P*-values for tests between effect sizes indicated at the top of the plot.


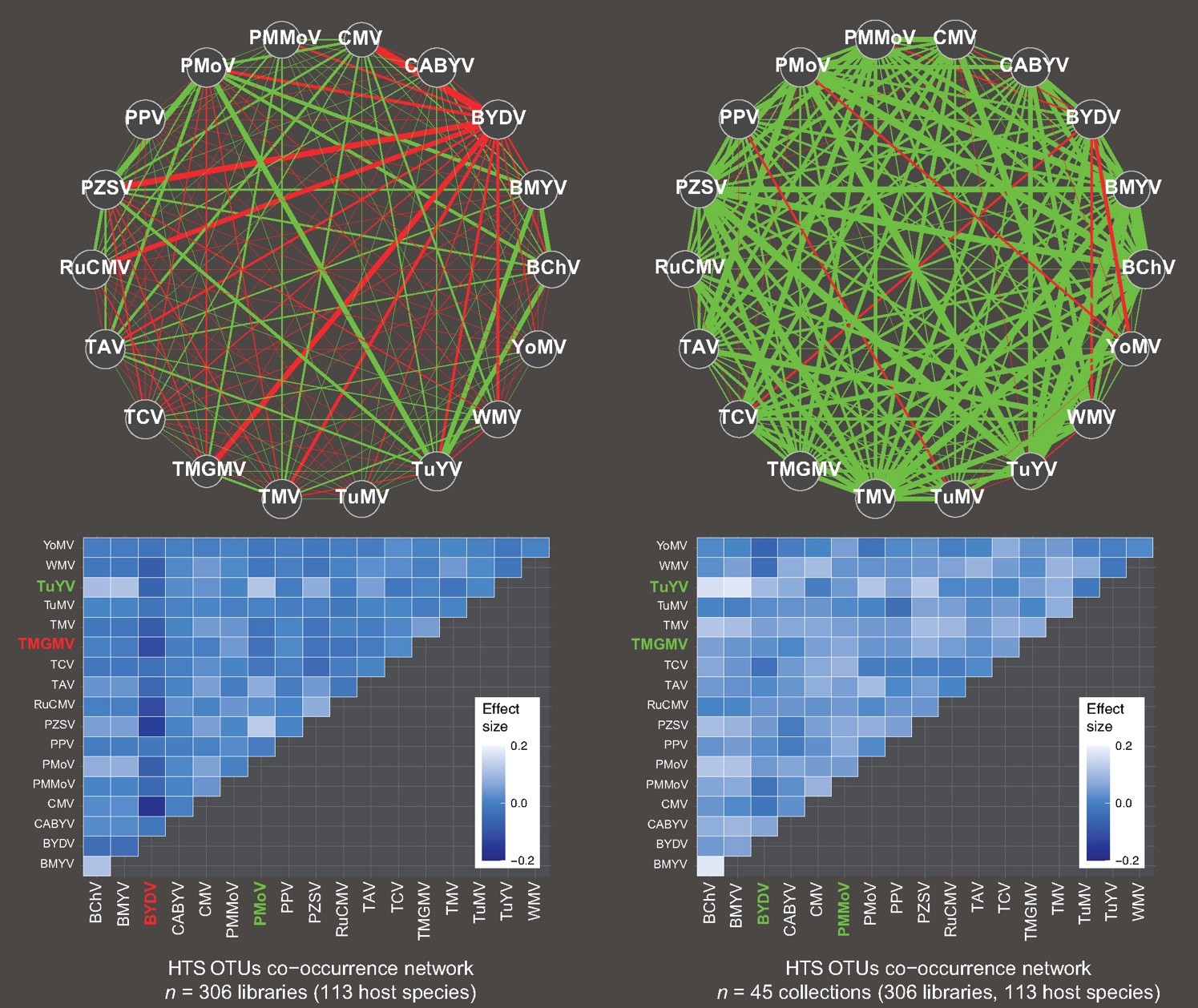


**Supplementary Figure 8.** Network and heatmap of effect sizes for the HTS2 dataset (left panel) aggregated by collection (right panel).
